# Fairness views and cooperation under varying levels of economic inequality

**DOI:** 10.1371/journal.pone.0288790

**Published:** 2023-11-09

**Authors:** Wasilios Hariskos, Jakob Neitzel, Lauri Sääksvuori

**Affiliations:** 1 Center for Empirical Research in Economics and Behavioral Sciences (CEREB), University of Erfurt, Erfurt, Germany; 2 Department of Economics, University of Hamburg, Hamburg, Germany; 3 Finnish Institute for Health and Welfare, Centre for Health Economics, Helsinki, Finland; 4 INVEST Research Flagship Center, University of Turku, Turku, Finland; Lund University: Lunds Universitet, SWEDEN

## Abstract

This paper investigates the impact of economic inequality on people’s perceptions of fairness and willingness to cooperate. Using experimental and survey data, we distinguish people’s injunctive perceptions of fairness from experimentally observed behavioral patterns. We find that impartial observers hold shared perceptions of fair contribution rules. Individuals with their own money at stake hold conflicting views over fair contribution rules. We find that contribution patterns are more scattered under strong inequality than under weak inequality. Overall, we observe that voluntary contributions are lower under strong inequality than under weak inequality. Our results contribute to the debate about the behavioral consequences of income and wealth inequalities in modern societies.

## 1. Introduction

Income, wealth, and consumption inequalities are pervasive features of human social organization. The sources of these inequalities are heavily studied and debated. Simultaneously, there is persistent debate about the socially acceptable levels of inequality. The scientific and political discussions of income and wealth inequalities have lately profited from the collection of historical data documenting the development of income and wealth distributions within and across societies over long time horizons. These data show that the number of people living in extreme poverty and economic inequalities between countries have decreased over the last decades [1, 2]. However, at the same time, income and wealth inequalities within societies have been strongly increasing since the 1970s across the developed world [3–6].

Accumulating evidence about the growth of income and wealth inequalities within societies has led to increasing concerns that large economic inequalities may undermine future economic growth and the long-term prosperity of human societies. There is intensive debate about the consequences of increasing income and wealth inequalities within societies on capital and labor markets [7, 8]. Moreover, economic inequalities are shown to be associated with a broad spectrum of health and social outcomes [9, 10]. However, characteristically, research aiming to understand the broader consequences of economic inequalities relies on cross-sectional income inequality and direct comparison of countries and states with varying levels of inequality. There is still limited knowledge about the causal effects of economic inequalities on the development of social capital, people’s perceptions of fair behavior, and willingness to cooperate. We do not know, for example, how economic inequality affects impartial observers’ views about socially appropriate choices (injunctive norms) in voluntary contribution situations and people’s behavior in sequential move voluntary contribution mechanisms.

This paper studies the causal effect of economic inequality on the emergence of fairness concerns and voluntary contributions to public goods. Using experimental and survey data, we study the effect of resource inequality on people’s perceptions of fairness and willingness to cooperate. Our controlled experiment and accompanying survey design enable to circumvent the challenges of causal inference related to observational studies on economic inequality. Moreover, using the combination of survey and experimental data, we can distinguish people’s injunctive perceptions of fairness from experimentally observed behavioral patterns. We expect that resource inequality between the decision-makers may raise varying views about fair contributions and define two behavioral rules relative to the contributions of other actors. First, we focus on the notion of *equality* that is generally thought of as the equalization of absolute contributions with no link to individual characteristics such as resources or capacity to contribute. Second, we focus on the notion of *equity* that links contributions to observable individual characteristics in a proportional manner and stipulates the equalization of relative contributions.

To complement the empirical evidence, we provide a simple theoretical model that embeds normative conflict over fair contribution rules into a utility framework by assuming that individuals derive utility from monetary income and adherence to normatively appealing contribution rules. Our model enables to organize some observed empirical regularities and suggests why less unequal groups may be more cooperative in sequential move public goods games than more unequal groups.

We find that impartial observers hold commonly shared (non-random) normative views of fair contribution rules. The most appealing injunctive contribution norm is the equity norm, despite differences in individual resources and levels of inequality. In the experiment, we observe that participants with high and low initial endowments hold conflicting views over fair contribution patterns. The observed contribution patterns are consistent with a self-serving interpretation of fairness. More uniquely, we find that observed contribution patterns are more dispersed under strong inequality than under weak inequality.

Our paper relates to a large body of literature that investigates the private provision of public goods. However, it is noteworthy that voluntary contributions to public goods seldom occur in isolation from the public production of public goods. Moreover, the state may facilitate the production possibilities of private public goods by altering property rights and economic incentives for voluntary contributions. Government policy to exempt charitable organization and donors from tax payments is a common example of a policy tool to facilitate the private provision of public goods.

Our work builds on and contributes to several strands of literature in economics and behavioral sciences. Here, we aim to relate our contribution mainly to the economics literature on inequality. Yet, we note that the associations between socio-economic status, income and generosity are currently heavily studied and debated across the social sciences. Several influential papers have argued that high income individuals are more selfish and less generous across multiple behavioral domains [11, 12]. However, there are substantial concerns about the research methods used to investigate these questions and accumulating empirical evidence to counter the conclusion that high socio-economic status predicts increased unethical behavior [13–17].

Literature about the consequences of economic disparities on people’s willingness to cooperate has for long relied on theoretical investigations. Meltzer and Richard [18] show that larger income inequality may result in greater public good provision, if the mean income simultaneously rises relative to the income of the median voter. In the same vein, Warr [19] and Bergstrom et al. [20] theoretically show that the total supply of public goods is unaffected by small redistributions of wealth. In an empirical study, Alesina and la Ferrara [21] use survey data on group memberships and data on U.S. localities to show that income inequality decreases the level of privately provided public goods in local communities.

More closely related to our contribution is the literature that studies the impact of endowment heterogeneity on public goods provision and cooperation in controlled experiments. The evidence from these experiments is mixed. Ostrom et al. [22], van Dijk et al. [23] and Cherry et al. [24] report evidence that contributions to public goods are lower in groups with heterogeneous endowments than in groups with homogenous endowments. Keser et al. [25] distinguish between weakly and strongly asymmetric initial endowments and find that total contributions are significantly lower in the strongly asymmetric treatment. Hargreaves-Heap et al. [26] control for variation in individual endowment sizes and find that the pure effect of endowment inequality on public goods provision is clearly negative. By contrast, Chan et al. [27] and Chan et al. [28] report that endowment heterogeneity increases voluntary contributions. Sadrieh and Verbon [29] find that neither the extent nor the skew of inequality affects cooperativeness and Hofmeyr et al. [30] find that randomly induced wealth heterogeneity affects neither the total contributions to public good nor the relative shares of individual contributions. Finally, Buckley and Croson [31] observe that less wealthy individuals often contribute same absolute amount than wealthier individuals.

It is noteworthy that a vast majority of experiments studying the effects of endowment inequality on cooperation are conducted using information conditions in which participants are informed about the size of asymmetric endowments. However, relevant to our study, Van Dijk and Grodzka [32] study the effect of endowment asymmetry and participants’ information about this asymmetry on actual and preferred contributions to a public good. Their results suggest that individuals informed about the asymmetry are more likely to prefer contributions that are proportional to the initial endowment, while uninformed individuals are more likely to prefer equal absolute contributions.

Our study differs in several ways from the existing experimental literature on the appeal of different fairness perceptions and voluntary public good provision. First, we study fairness and voluntary public good provision using a sequential decision structure. The sequential order of decisions is apparent in many naturally-occurring voluntary contribution decisions varying from group work at schools and universities to large-scale fund-raising campaigns that regularly announce prior contributions in a sequential order. The sequential order structure enables us to examine how individuals endowed with unequal endowments condition their behavior on the observed contributions of other individuals. Second, we induce endowment heterogeneity between participants using a real-effort tournament. This practice can be expected to shift contribution patterns from simple egalitarianism towards the equalization of relative contributions and enhance the prominence of conflicting normative ideals over fair contribution rules compared to a random allocation of initial endowments [33]. Third, we elicit participants’ injunctive and descriptive perceptions of fairness and measure their willingness to cooperate exclusively in the presence of endowment inequality, enabling us to examine the appeal of multiple plausible fairness principles under varying levels of inequality. Consequently, we circumvent the problem that the notions of equality (equal absolute contributions) and equity (equal relative contributions) overlap in homogenous wealth distributions.

Our empirical results are largely consistent with the literature documenting that larger endowment inequality decreases willingness to cooperate in public goods games. Likewise, our results are consistent with the literature that has documented self-serving bias in fairness judgements and behaviors [e.g., 34, 35]. However, our study provides a more nuanced picture about the effects of resource inequality on injunctive and descriptive views over fair contribution rules. Our primary contribution is to simultaneously document the effect of increasing economic inequality on injunctive and descriptive views over fair contributions. Moreover, we document an association between the erosion of commonly shared contribution patterns and low levels of cooperation under strong economic inequality. Overall, our results reinforce the view that larger economic inequalities within human groups may lead to less cooperative behaviors.

## 2. Decision setting

### 2.1 The voluntary contribution mechanism

We study voluntary public good provision in a population of *n* ≥ 2 players. There are two distinct types of players: rich players with an endowment *w*_*R*_ and poor players with an endowment *w*_*P*_, where *w*_*R*_>*w*_*P*_. We use the Voluntary Contribution Mechanism (VCM) such that after individual contribution decisions player *i* receives income determined by

$$\pi_{i}(c_{i},c_{-i})=w_{i}-c_{i}+\alpha (c_{i}+c_{-i})$$

where $\frac{1}{n}<\alpha <1$ is the marginal per capita return on contributions, *w*_*i*_∈{*w*_*R*_, *w*_*P*_} is player *i*’s endowment, *c*_*i*_ is his or her own contribution to the public good and *c*_−*i*_ = ∑_*j*≠*i*_*c*_*j*_ are the contributions of all players except *i*. Since every unit contributed to the public good generates *α* additional units for all members of the group, it is socially optimal for everyone to contribute their entire endowment to the public good. However, since the individual return from contributions is smaller than one, the unique subgame perfect equilibrium with own-payoff maximizing individuals is zero contributions (free-riding equilibrium).

### 2.2 The plurality of fairness principles

The presence of endowment heterogeneity between the players suggests the focality of multiple contribution rules. These rules can conceptually be distinguished into two dimensions: efficiency and fairness [36]. An efficiency rule suggests maximal contributions by all to achieve the socially optimal level of contributions, while fairness rules prescribe contributions that are relative to the contributions of other actors. This paper largely leaves aside the tension between efficiency and fairness and focuses on two prominent fairness rules that prescribe contributions relative to the contributions of other actors.

We extend the analysis of voluntary public good provision beyond the pure own-payoff maximization and assume that agents value utility derived from monetary income and adherence to normative principles of fair contributions. The notion of *equality* suggests the equalization of absolute contributions with no relation to individual characteristics such as the initial endowment. By contrast, the notion of *equity* suggests that fair contributions are linked to individual characteristics such as the initial endowment in a proportional manner and stipulates the equalization of relative contributions to the public good.

Our model characterizes a situation in which players face two distinct types of decisions. First, players choose their preferred contribution principle. We assume that individuals choose the contribution principle which yields the highest utility for the player type. Second, players contribute to the public good given their type-specific utility function that contains their preferred contribution principle. Following the utility function proposed by Cappelen et al. [37], we assume that player *i*’s decision problem subject to constraints can be summarized as:

$$\underset{c_{i},k}{\max}\;u_{i}(c_{i},c_{-i},k)=\pi_{i}(c_{i},c_{-i})-\frac{\beta }{2}{\left(c_{i}-m(k,c_{-i})\right)}^{2}s.t.c_{i}\in [0,w_{i}]\&k\in \{0,1\},$$

where the parameter *β*≥0 determines the weight that is attached to deviations from the applied contribution principle *m*(*k*, *c*_−*i*_). The contribution principle depends on the applied principle *k* and the contributions of other players. It is defined as

$$m(k,c_{-i})={\left(\frac{1}{n-1}\right)}^{1-k}{\left(\frac{w_{i}}{w_{-i}}\right)}^{k}c_{-i},$$

where *w*_−*i*_ = ∑_*j*≠*i*_*w*_*j*_ denotes the sum of other players’ initial endowments. Hence, if *k = 0*, the choice of contribution principle implies that a player uses the absolute contributions of other players as a reference point with no relation to initial endowments and is said to apply the equality principle, since $m(0,c_{-i})=\frac{1}{n-1}c_{-i}$. By contrast, if *k = 1*, the choice of contribution principle implies that a player accounts for the differences in initial endowments $m(1,c_{-i})=\frac{w_{i}}{w_{-i}}c_{-i}.$

Our primary interest pertains to the selection of alternative contribution principles and impact of varying levels of endowment inequality in sequential voluntary contribution mechanisms. In a sequential contribution mechanism, we separate players into first- and second-movers. A natural alternative to this two-stage structure would be to let all players move sequentially. However, a fully sequential structure is likely to put the main emphasis on individual first-movers as leaders, which is not the focus of this paper. Using a two-stage structure and groups of simultaneously moving players at each stage has the advantage of focusing on different type of actors rather than on individual leaders. Moreover, an experimental design with multiple first-movers enables to test whether second-movers condition their behavior more strongly on the average or minimum first-mover contributions.

In conjunction with endowment heterogeneity, a two-stage structure leads to three different combinations of first- and second-movers: (a.) all rich players contribute first, (b.) all poor players contribute first, or (c.) rich and poor players contribute in a mixed order such that there is at least one first- and second-mover of each type.

### 2.3 Conjectures

We formulate two empirically testable conjectures using the above-described theoretical framework. First, we study the selection of preferred normative principles of second-movers and derive the following conjecture.

**Conjecture 1** *Rich players prefer the equality principle*, *whereas poor players prefer the equity principle*.

The intuition behind the conjecture is straightforward. For rich players, adherence to the equality principle requires lower contributions than adherence to the equity principle $(\frac{c_{-i}}{n-1}\leq \frac{c_{-i}}{w_{-i}}w_{i},\;since\;w_{i}>\frac{w_{-i}}{n-1}).$ For poor players, the opposite applies as the equity principle stipulates lower contributions than the equality principle $(\frac{c_{-i}}{w_{-i}}w_{i}\leq \frac{c_{-i}}{n-1},\;since\;w_{i}<\frac{w_{-i}}{n-1}).$ A complete proof of the conjecture is provided in the Online Appendix E in S1 Appendix.

In the following analysis, we assume that all second-movers contribute according to their preferred normative principle after observing the contributions by first-movers. Thus, rich second-movers use the average absolute amount contributed by first-movers as their normative principle, while poor second-movers use the average amount contributed by first-movers relative to the initial endowments of first-movers as their normative principle. Formally, $c_{i}=\frac{c_{fm}}{{\#}_{fm}}$, if player *i* is a second-mover and has an endowment *w*_*R*_, and $c_{i}=\frac{c_{fm}}{w_{fm}}w_{i}$, if player *i* is a second-mover and has an endowment *w*_*P*_. Here, *c*_*fm*_ denotes the sum of first-mover contributions, #_*fm*_ denotes the number of first-movers and *w*_*fm*_ is the sum of first-mover endowments.

The same reasoning is not applicable to first-mover contributions as contribution decisions cannot be based on any observed contributions by other players. Thus, we assume that first-movers employ a simple heuristic to determine their contribution level and contribute a fixed proportion, *0 ≤ x ≤ 1*, of their endowment, if the resulting contribution is not larger than the endowment of the poor players, in which case their contribution is *w*_*l*_. Hence, *c*_*i*_ = min{*xw*_*i*_, *w*_*l*_}, if player *i* is a first-mover. Consequently, we define a group to be subject to weak inequality if *xw*_*h*_≤*w*_*l*_ and to strong inequality if *xw*_*h*_>*w*_*l*_.

**Conjecture 2** *Contributions to the public good are lower under strong inequality than under weak inequality independent of the sequential order of contributions*.

We provide an intuition for the conjecture using the mechanism with rich first-movers. Notably, as long as the group operates under weak endowment inequality, the level of public good provision is *x*∑_*i*_*w*_*i*_. However, once the threshold to strong endowment inequality is transcended, rich players contribute a smaller share of their endowment. As a reaction to this, poor individuals lower their contributions. Contributions are now based only on the endowment of the poorer type and increasing endowment inequality lowers the total amount of contributions. We provide a complete proof for the conjecture in the Online Appendix E in S1 Appendix.

## 3. Experimental and survey design

### 3.1 Experimental design

We conducted a laboratory experiment to study contribution patterns and voluntary cooperation under weak and strong endowment inequality. The experiment consisted of two parts. The first part was a real-effort task. We used this task to determine initial endowments. We used the Encryption Task [38] which proceeded as follows. Participants were divided into groups of four and received an encryption table which assigned a number to each letter of the alphabet. During the following ten minutes, participants were presented words which needed to be encrypted by substituting the letter with numbers using the encryption table. The words were presented in a predetermined sequence. A participant could not proceed to the next word before the current word was correctly encrypted. The two group members with the highest numbers of encrypted words received a high initial endowment. The two other group members received a low initial endowment. Potential ties in the rank order were broken at random.

Participants stayed in the same group of four throughout the experiment and learned at the beginning of the second part if they were among the two most productive individuals in the group in the first part. However, we did not reveal the exact number of encrypted words by participant. After the completion of the real-effort task, participants received new experimental instructions for the second part.

The second part of the experiment consisted of a sequential public goods game with two first-movers who contributed simultaneously and two second-movers who contributed simultaneously after having observed the first-mover contributions. We used a standard linear public goods game where the monetary payoff of player *i* was determined by

$$\pi_{i}(c_{i})=w_{i}-c_{i}+0.4*C,$$

where *w*_*i*_ is *i*’s endowment, *c*_*i*_ is *i*’s contribution and *C* is the sum of contributions by all four players. The public goods game was played in fixed groups for 15 consecutive periods.

Our experimental design consisted of four treatments that vary the degree of resource inequality and sequence of play. We implemented two treatments where rich individuals contributed first and two treatments where poor individuals contributed first. Table 1 provides an overview of the 2×2 experimental design.

**Table 1:** Experimental treatments.

	WEAK inequality {*w*_*R*_ = 25 & *w*_*P*_ = 15}	STRONG inequality {*w*_*R*_ = 30 & *w*_*P*_ = 10}
Rich first-movers	WEAK-RICH	STRONG-RICH
Poor first-movers	WEAK-POOR	STRONG-POOR

We kept overall resources constant at *W* = *w*_*R*_+*w*_*P*_ = 40 in all treatments. In the two WEAK treatments, rich players received an endowment of 25 Experimental Currency Units (ECUs) and poor players an endowment of 15 ECUs. In the two STRONG treatments, the endowments were 30 ECUs for the rich players and 10 ECUs for the poor players. After each period, participants were informed about the sum of contributions in their group, the exact contribution of every individual in the group and the initial endowment of the individual. To track individual behavior during the experiment, we assigned a unique identification letter (A, B, C or D) for every participant and listed individual contributions always in the same order. We assigned for the two first-movers letters A and B and for the two second-movers letters C and D.

In addition to the contribution decisions, we elicited participants’ beliefs about the behavior of other group members in the current period. We asked participants to predict the contribution of the same participant type than they are and the average contribution of two other type of participants. Participants were paid a small reward based on the accuracy of their estimates.

### 3.2 Experimental procedure

We conducted the experiment at the experimental laboratory of the School of Business, Economics and Social Sciences at the University of Hamburg. At the University of Hamburg ethical review is standardized for conventional socioeconomic experiments such as this one. This implies that the treatment of participants agreed with the RESPECT code of practice for socioeconomic research in Europe by the European Commission and the guidelines of the German Research Foundation (Deutsche Forschungsgemeinschaft). All participants gave their written informed consent to participate voluntarily, assuring them that analyses, and the experimental data would be published without an association to their real identities. Moreover, random assignment to visually separated cubicles and private payment at the end of the experiment preserved the anonymity of participants. The experiment involved no deception of participants. As in other socioeconomic experiments, there were no additional ethical concerns.

For programming the experiment, we used z-tree [39]. For recruiting the participants, we used ORSEE [40] and H-Root [41]. We ran eight different sessions with 192 subjects. The vast majority of 88 female (Age Mean: 24.3, Std: 2.98, Min: 18, Max: 38) and 104 male (Age Mean: 25.2, Std: 4.19, Min: 19, Max: 38) subjects were undergraduate students. At the beginning of each session, the experimental team randomly assigned participants to their cubicles. To ensure common knowledge, participants received written instructions and a member of the research team read them aloud. Thereafter, participants took a post instruction quiz and were not allowed to continue until all answers were correct. All decisions were made privately. At the end of the experiment, we chose one of the 15 periods at random to determine the earnings from the public goods game. The sessions lasted approximately 75 minutes including instructions, post instruction quiz, demographic questionnaire, and payment procedure. Earnings per participant were on average €13.43. At the time of data collection, the median gross hourly wage in Germany amounted to €15.50 [42]. The typical hourly wage for participants, mainly undergraduate students, outside of the experiment ranged from €8 to €11 [42].

The full experimental data set includes two additional treatments that are not reported in this paper. These data are described in an earlier working paper [43] and include a treatment with a simultaneous move structure (weak inequality) and a treatment with a sequential move structure in which rich and poor individuals contribute in a mixed order (weak inequality). We chose to not report findings from these two treatments to focus on the effect of economic inequality on contribution patterns and cooperativeness in a sequential move voluntary contribution mechanism. An English translation of the original experimental instructions (in German) is available in the Online Appendix F in S1 Appendix. All collected data and replication codes needed to reproduce all research findings reported in this paper are available at the Open ICPSR data repository—openicpsr-189521 | https://doi.org/10.3886/E189521.

We assess the success of our randomization procedure and report in Table 2 the number of encoded real-effort tasks by treatment and gender. As expected, due to the randomization, there are no differences in the number of encoded tasks between treatments (Kruskal-Wallis test between the four treatments, *χ*^*2*^*(3) = 2*.*951*, *p = 0*.*399*). There are no differences in the number of encrypted words between men and women (Number of encoded words by Men = 44.9 and by Women = 43.9, Two-sample t-test, *t = 0*.*710*, *p = 0*.*479*). Thus, there are also no gender differences in the composition of high and low endowed participants (Fisher’s exact test, *p = 0*.*885*). In the same vein, Online Appendix C (Fig A2) in S1 Appendix shows that there is no clear association between relative performance in the real-effort task within the group of rich and poor participants and contributions in the experiment. This observation is consistent with the fact that our experimental design informed participants only about their placement among the two top encoders in their group but did not reveal the exact number of encrypted tasks by other participants.

**Table 2:** Number of encoded tasks by treatment and gender.

Treatment	Variable					
	Encoded				High endowment	N
	(Mean)	(Std.)	(Min)	(Max)	(%)	
WEAK-RICH	44.9	9.8	24	68	-	48
Male	46.7	9.4	29	68	54.2	24
Female	43.1	10.1	24	58	45.8	24
WEAK-POOR	42.5	9.6	27	65	-	48
Male	41.9	9.5	27	63	47.8	23
Female	43.1	9.7	28	65	52.0	25
STRONG-RICH	45.2	8.3	28	67	-	48
Male	45.0	9.0	28	67	46.4	28
Female	45.5	7.4	34	57	55.0	20
STRONG-POOR	45.1	8.2	32	64	-	48
Male	45.5	7.3	33	64	48.3	29
Female	44.4	9.5	32	59	52.6	19

### 3.3 Survey design

In addition to the incentivized experiment, we conducted an online survey among external impartial observers to elicit their views about fair contributions in the above-described public goods game. To compare the incidence of alternative views of fair contributions between experimental participants and impartial external observers, we conducted the online survey using the same pool of participants and recruitment methods (e-mail invitation) than in the experiment. However, using the online recruitment system, we excluded from the survey study individuals who had previously participated in the experiment.

The online survey began with the participants reading the full instructions of the experiment, including the section that described the real-effort task used in the experiment. Thus, the external observers were aware that the starting endowments in the experiment were generated through participation in a real-effort tournament. Thus, to our knowledge, this paper reports the first results from a survey to study uninvolved individuals’ views of fair contribution after inducing heterogenous endowments using a real-effort tournament.

There were 100 participants in the survey study. There were 40 female (Age Mean: 24.5 Std: 3.1, Min: 20, Max: 32) and 60 male (Age Mean: 25.8, Std: 5.65, Min: 17, Max: 55) participants who were mostly undergraduate students from various academic disciplines. Two randomly drawn participants were awarded with a 100€ cash prize for completing the questionnaire.

Every participant in the survey study answered the same set of questions. The complete set of questions included two scenarios that varied the initial distribution of endowments (weak vs. strong inequality). To control for possible order effects, the order of different scenarios and the order of questions within each scenario was randomized. In all scenarios, we asked participants to answer two different types of questions. First, we elicited their unconditional view about fair contributions and asked participants to answer the following question: “From the viewpoint of a neutral external observer, what is in your opinion a fair contribution to the group account by a group member who has an endowment of 25 (15) ECUs?”. Second, we elicited their views about fair contributions using a conditional question and asked participants to answer the following question: “From the viewpoint of a neutral external observer, what is in your opinion a fair contribution to the group account by a group member who has an endowment of 25 (15) ECUs, if a group member with 15 (25) ECUs has contributed 9 ECUs to the group account?”. The reference contribution of 9 ECUs in conditional questions was chosen to maximize the number of alternative fairness principles that coincide with integer contributions. Like many experimental public goods games, our experimental design allowed only integer contributions. There was no explicit restriction to use only integer numbers in the survey. The exact contribution levels that coincide with alternative contribution norms are shown in the Online Appendix A in S1 Appendix using strict categorization rules (Table A1) in S1 Appendix and using categorization rules that allow small decision errors (Table A2) in S1 Appendix.

Our survey design builds on two related studies. Reuben and Riedl [36] conduct a related questionnaire study about the normatively appealing contribution rules among impartial individuals in simultaneous move public goods games. Nikiforakis et al. [44] elicit normatively appealing rules of behavior among their participants after participation in an experiment. Our questionnaire study closely follows the procedure developed by Reuben and Riedl [36].

The contribution of our survey study is threefold. First, our data complement previous studies and suggest how contextual differences between our study and the study by Reuben and Rield [36] translate into differences in normative views of fair contributions. Second, we obtain evidence about the normative views of fair contribution rules among impartial and partial individuals. This enables us to investigate the potential discrepancy between the injunctive perceptions of fairness and observed contribution patterns. Third, the survey data is used to collect direct evidence about the prominence of multiple normative principles among external observers and evaluate the potential limitations of our theoretical and empirical approaches that focus on the notions of equality and equity. Overall, there are multiple plausible normative principles beyond equality and equity that could be relevant in the public goods game (e.g., equality of earnings and efficiency). To provide a more comprehensive picture about participants’ perceptions of fairness and evaluate the limitations of our empirical approach, we report in the Online Appendix A (Table A3) in S1 Appendix the fraction of survey answers that correspond with the equity, equality, efficiency, and equal payoff principles.

## 4. Results

This section summarizes our main empirical findings. First, we present descriptive statistics for the survey data. Second, we characterize the most appealing contribution rules among the impartial external observers. We interpret these observations as injunctive fairness principles that fairness minded individuals ought to follow in the experiment. Third, using experimental data, we contrast the injunctive fairness principles with observed behavioral patterns. Finally, we investigate the average levels of cooperation under weak and strong inequality.

### 4.1 Injunctive contribution norms

**Fig 1:**
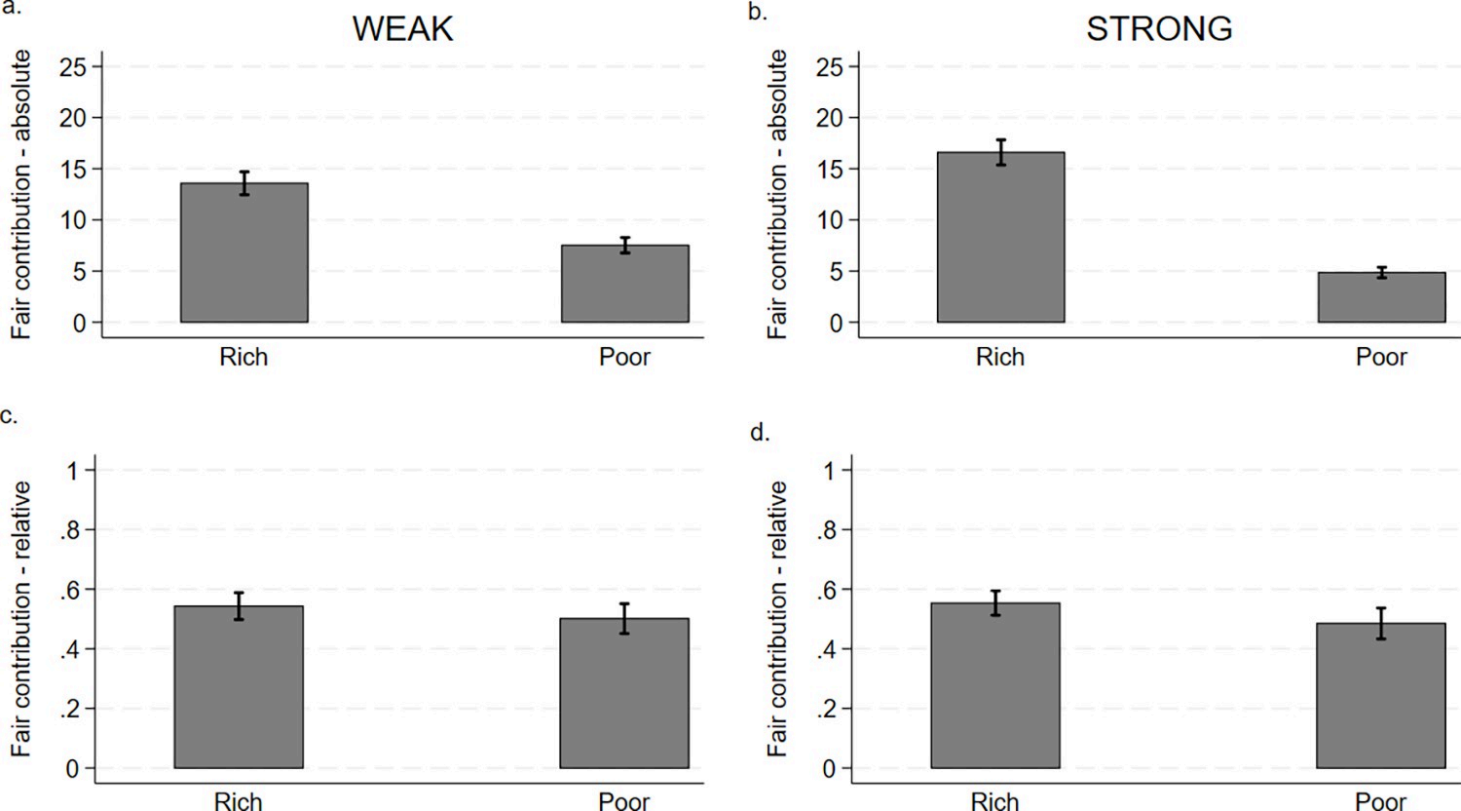
Third-parties’ average opinions (95% confidence intervals) about fair absolute and relative contributions by rich and poor participants under weak and strong inequality.

Fig 1. presents how many ECUs do third-parties believe rich and poor participants should contribute under weak and strong inequality. Fig 1(A) plots third-parties’ average opinions about fair absolute contributions under weak inequality, while Fig 1(B) plots third-parties’ average opinions about fair absolute contributions under strong inequality. Fig 1(A) and 1(B) suggest that rich participants should contribute substantially more in absolute terms than poor participants under weak and strong inequality. Fig 1(C) and 1(D) document that the suggested contributions equal approximately half of the initial endowments under weak and strong inequality. Taken together, Fig 1 provides descriptive evidence that the third-parties’ average opinions align with equal relative contributions of the endowment.

**Fig 2:**
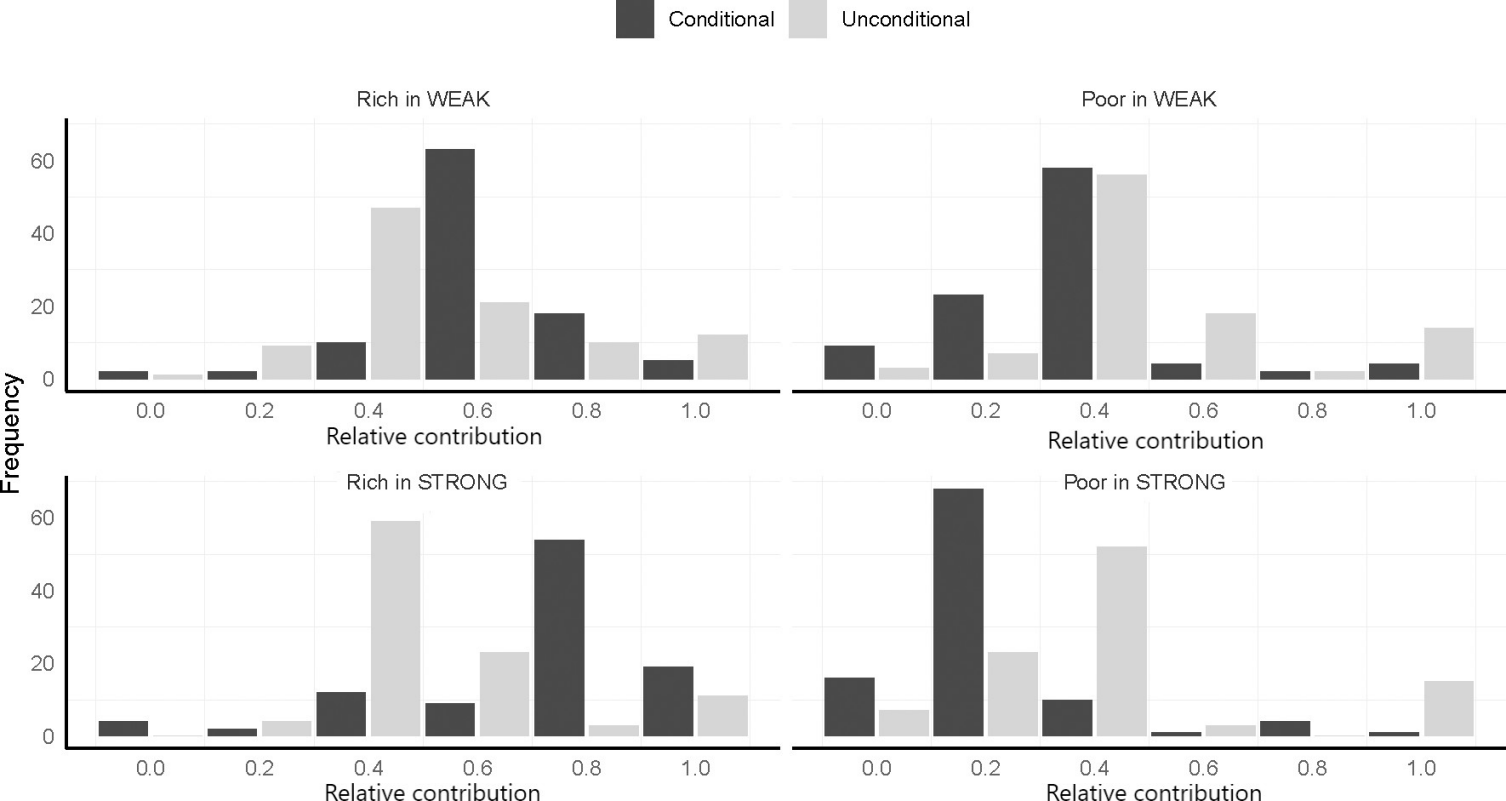
Histograms of answers to the conditional and unconditional survey questions.

Fig 2. presents the distributions of suggested unconditional and conditional contributions by player type and treatment. We observe that survey respondents’ answers are concentrated at a few focal values. To quantify the significance of this observation, we perform one-sample chi-square tests for all (eight) distributions shown in Fig 2 against a uniform distribution and reject the hypothesis that the observations are drawn from a random uniform distribution in all cases (*p* < 0.001). Thus, we find that the respondents appear to have some (non-random) mutual understanding about fair contributions to the group account.

In the following, we quantify the normative appeal of alternative contribution principles among the impartial external observers. We compute for every impartial observer contribution ratios using their answers to the unconditional and conditional survey questions. To ensure comparability between the contribution ratios, we always keep rich participants’ contributions in the numerator and define alternative contribution ratios as follows. The unconditional contribution ratio is the stated fair contribution level for rich individuals divided by the stated fair contribution level for poor individuals. Consequently, we can compute one unconditional contribution ratio per participant under weak and strong inequality.

Using participants’ answers to conditional survey questions, we can compute separate conditional contribution ratios for rich and poor individuals under weak and strong endowment inequality. Let *s* be the fair contribution level stated by the impartial observer. The conditional contribution ratio of poor to rich individuals is $R=\frac{9}{s}$, where *s*∈{0,1,…,*W*_*P*_}. The contribution ratio of rich to poor individuals is $R=\frac{s}{9}$, where *s*∈{0,1,…,*W*_*R*_}. A ratio of *R = 1* defines the equality principle (equal absolute contributions) in all treatments and a ratio of *R = 1*.*66 (R = 3)* defines the equity principle (equal relative contributions) under weak (strong) resource inequality. Moreover, we note that at the extreme, contributions lead to the following corner cases. For the contribution ratio of poor to rich individuals, as *s → 0*, then *R → ∞*, and the contribution ratio approaches perfect selfishness. For the contribution ratio of poor to rich individuals, as *s*→*W*_*P*_, then $R=\frac{9}{W_{P}}$, and the contribution ratio approaches perfect altruism and efficiency. For the contribution ratio of rich to poor individuals, as *s*→0, then *R*→0, and the contribution ratio approaches perfect selfishness. For the contribution ratio of rich to poor individuals, as *s*→*W*_*R*_, then $R\to \frac{W_{R}}{9}$, and the contribution ratio approaches perfect altruism and efficiency.

**Fig 3:**
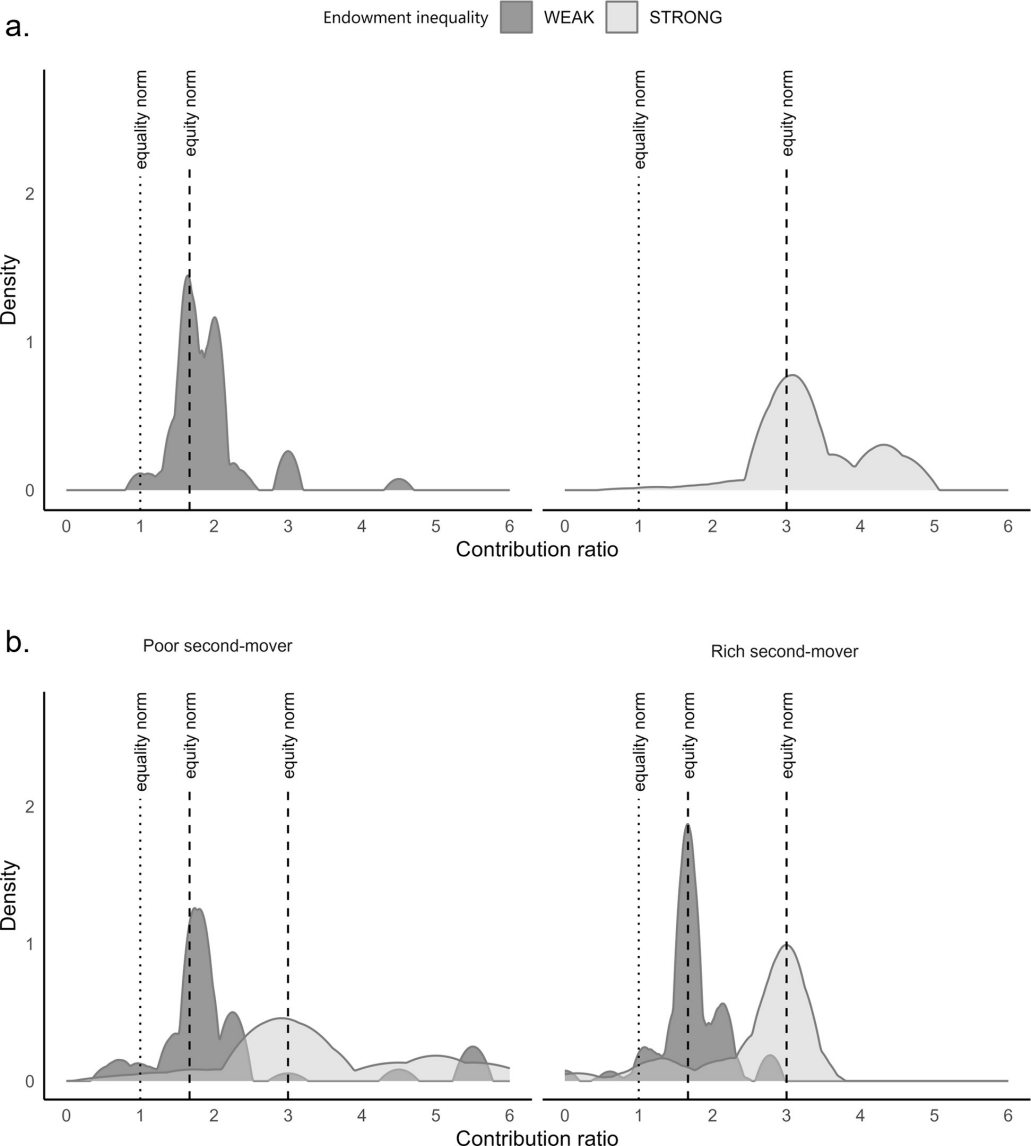
The distributions of suggested contribution ratios by individual type and level of endowment inequality. Figure shows Kernel densities of average contribution ratios using Epanechnikov kernel functions. Bandwidths are calculated to minimize the mean averages squared error for the underlying Gaussian density.

Fig 3(A) plots the distributions of suggested unconditional contribution ratios under weak and strong endowment inequality. We observe that the distributions are more likely to be concentrated around the equity principle than around the equality principle (Weak inequality: Average absolute deviation from the equity principle = 0.30, Average absolute deviation from the equality principle = 0.86, Wilcoxon signed-rank test, *z = -7*.*646*, *p < 0*.*001*; Strong inequality: Average absolute deviation from the equity principle = 0.44, Average absolute deviation from the equality principle = 2.27, Wilcoxon signed-rank test, *z = -8*.*881*, *p < 0*.*001)*. It is noteworthy that the distribution of unconditional contribution ratios under weak endowment inequality has a secondary peak at R = 2 that does not correlate with any clearly defined contribution principle. A more detailed analysis of unconditional survey questions (Online Appendix D, Table A4 in S1 Appendix) shows that a substantial number of respondents report that the fairest combination of contributions under weak endowment inequality is 10 ECUs by rich participants and 5 ECUs by poor participants. This reported combination of contributions may have served as a simple approximation of the equity principle. Overall, Fig 3(A) shows that, based on unconditional contribution ratios, there is little support for the equal absolute contributions principle among the impartial external observers.

Fig 3(B) plots the distribution of conditional contribution ratios by player type and level of endowment inequality. We make two observations. First, all distributions have a clear peak. These peaks coincide more with the equity principle than with the equity principle, despite differences in individual endowments and the level of endowment inequality. We quantify the statistical significance of this observation using a following procedure. We compute contribution ratios and their absolute deviations from the equity and equality principles for all individuals and use Wilcoxon signed-rank test to examine if the contribution ratios are closer to the equity principle than to the equality principle. Overall, as evidenced in Table 3, we find little support for equal absolute contributions among the impartial external observers.

**Table 3:** Average absolute deviations from equal absolute and relative contributions.

Survey measure	Average Deviation | Contribution ratio–contribution principle |		P-value
	Equity	Equality	
Weak inequality–Rich move first	0.43	0.90	< 0.001
Weak inequality–Poor move first	0.29	0.76	< 0.001
Strong inequality–Rich move first	0.53	2.25	< 0.001
Strong inequality–Poor move first	0.52	1.64	< 0.001

Notes: P-values are based on two-sided Wilcoxon signed-rank tests

Second, the distributions are more concentrated around the equity principle under weak inequality than under strong inequality (Average absolute deviation from the equity norm: Weak inequality = 0.32, Strong inequality = 0.48, Wilcoxon signed-rank test, *z = -3*.*056*, *p = 0*.*002*). This observation suggests that individuals hold more scattered normative views over fair contribution rules under strong inequality than under weak inequality. Overall, we observe that the most appealing contribution rule is the equity norm.

**Result 1** *Impartial observers hold shared perceptions of fair contribution rules*. *The most appealing injunctive contribution norm is the equity norm*. *Contribution rules are more scattered under strong inequality than under weak inequality*.

### 4.2 Observed contribution patterns

In the following, we investigate how survey respondents’ perceptions about fair contributions are related to contribution patterns elicited using experimental data. We compute for every second-mover a contribution ratio based on participant’s own contribution and the average contribution by the two first-movers. We compute the average contribution ratio over the 15 periods at the group level to obtain one contribution ratio per group. Analogously to the previously defined contribution ratios, a ratio of *R = 1* implies equality principle as a central tendency. A ratio of *R = 1*.*66 (R = 3)* implies equity principle as a central tendency under weak (strong) wealth inequality.

**Fig 4:**
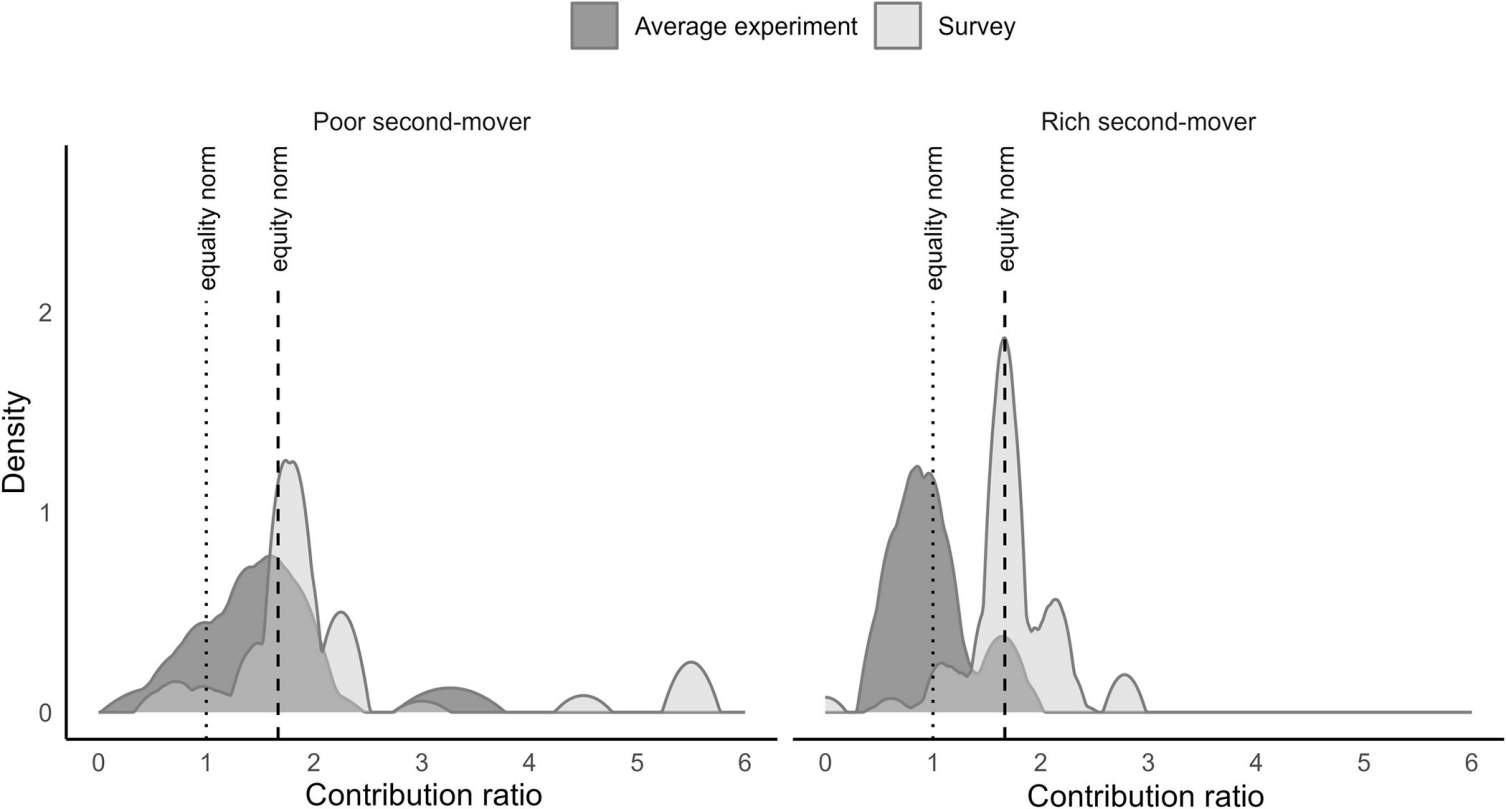
The distribution of observed contribution ratios by poor and rich individuals under weak endowment inequality. Figure shows Kernel densities of average contribution ratios using Epanechnikov kernel functions. Bandwidths are calculated to minimize the mean averages squared error for the underlying Gaussian density.

Fig 4 plots the distribution of most appealing contribution ratios among the impartial external observers and the distribution of contribution ratios by experimental participants under weak wealth inequality. The distribution of most appealing contribution ratios among the impartial external observers is based on their answers to the conditional norm elicitation question. The contribution ratios of experimental participants are computed at the group level.

We make two observations from Fig 4. First, the density distributions that plot the contribution ratios of experimental participants have clear peaks at *R = 1* and at *R = 1*.*66*. This observation suggests that participants hold conflicting normative views of fair contribution rules. Second, the preferred normative principles differ between participant types. We test the equality of distributions between the rich and poor individuals and reject the hypothesis that the distributions are equal (Kolmogorov-Smirnov test for the equality of distribution functions using group level observations, *d* = 0.667, *p* = 0.005). The peak of the density distribution among the poor participants coincides with the equity norm. By contrast, the peak of the density distribution among the rich participants coincides with the equality norm. Thus, the revealed contribution pattern of rich individuals does not only deviate from the revealed contribution pattern of poor individuals but from the normative principle preferred by the impartial external observers. Overall, we observe that experimental participants hold conflicting views over fair contributions. These views are consistent with a self-serving interpretation of fairness principles.

We complement these analyses by computing contribution ratios for experimental participants: (i) using only first period observations, and (ii) observations where the first-mover average contribution is 8–10 (9 ± 1) ECUs. Moreover, our experimental design enables us to report contribution ratios based on first-mover minimum contributions and test whether second-movers condition their behavior more strongly on the average or minimum first-mover contributions. We find that our results are robust to using only first period observations (Online Appendix C, Fig A6 in S1 Appendix), observations where the first-mover average contribution is 8–10 (Online Appendix C, Fig A7 in S1 Appendix) and first-mover minimum contributions instead of first-mover minimum contributions (Online Appendix C, Figs A8 and A9 in S1 Appendix). Results reported in Online Appendix D (Table A6) in S1 Appendix suggest that second-movers are equally likely to condition their behavior on the average and minimum contribution by the first-movers.

**Result *2*** *Rich and poor participants have conflicting views over fair contribution rules in treatments with weak endowment inequality*. *Observed contribution rules are consistent with a self-serving interpretation of fairness rules*.

**Fig 5:**
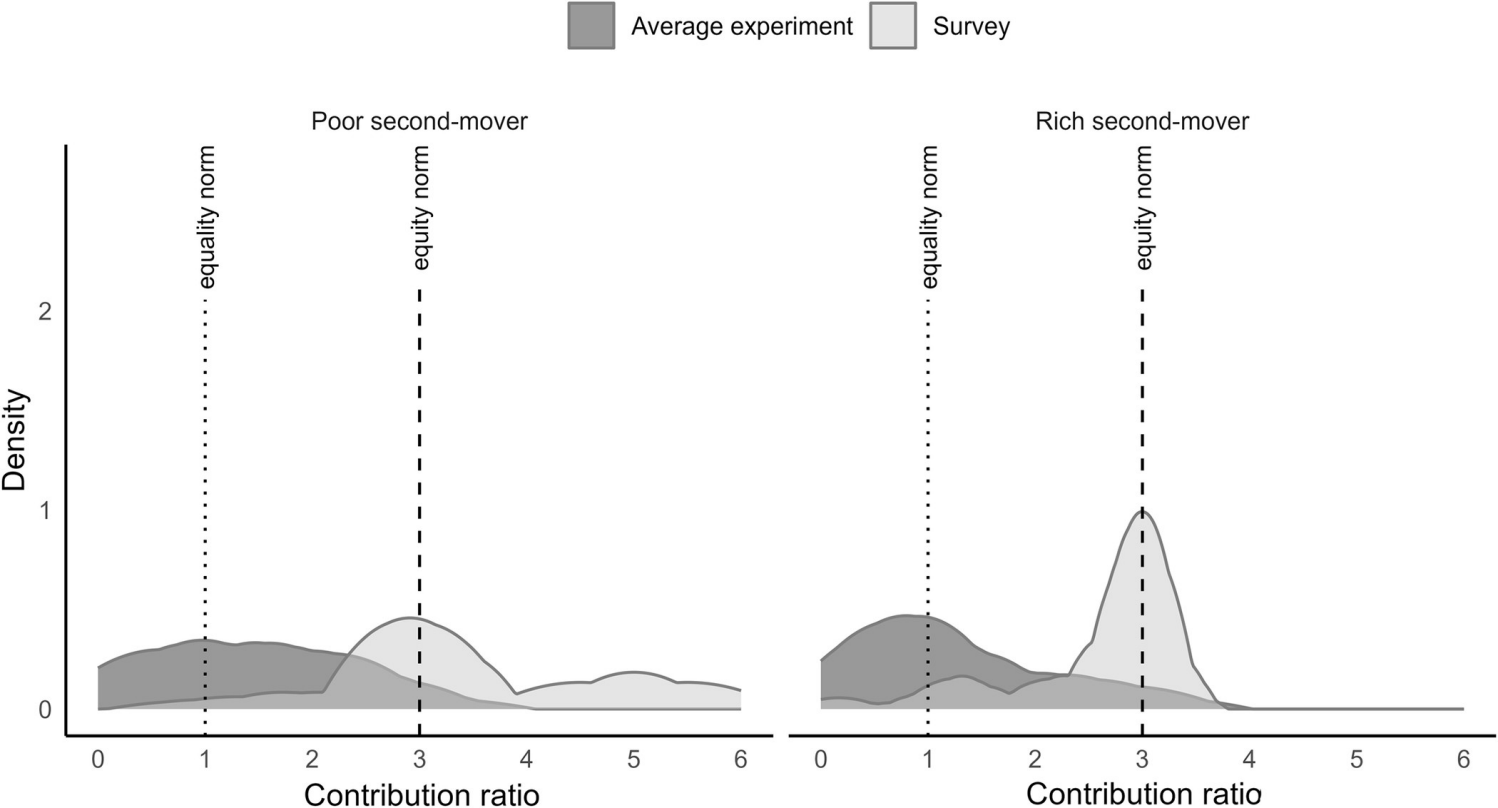
The distribution of observed contribution ratios by poor and rich individuals under strong endowment inequality. Figure shows Kernel densities of average contribution ratios using Epanechnikov kernel functions. Bandwidths are calculated to minimize the mean averages squared error for the underlying Gaussian density.

Fig 5 plots the distribution of most appealing contribution ratios among the impartial external observers and the distribution of contribution ratios revealed by the experimental participants under strong wealth inequality. We make two observations from Fig 5. First, the revealed contribution patterns of rich and poor participants align under strong wealth inequality. We test the equality of distributions between the rich and poor individuals and cannot reject the hypothesis that the distributions are equal (Kolmogorov-Smirnov test for the equality of distribution functions using group level observations, *d = 0*.*250*, *p = 0*.*472*). Second, a visual comparison of Figs 4 and 5 suggests that contributions are more dispersed under strong endowment inequality than under weak endowment inequality. To quantify the dispersion of contributions under weak and strong endowment inequality, we compute coefficients of variation by treatment and find that the coefficients of variation are substantially lower in treatments with weak endowment inequality than in treatments with strong endowment inequality (WEAK-RICH: *C*_*v*_ = 0.84, WEAK-POOR: *C*_*v*_ = 1.06, STRONG-RICH: *C*_*v*_ = 1.37, STRONG-POOR: *C*_*v*_ = 1.44, two-sided Wilcoxon rank-sum test for WEAK treatments vs. STRONG treatments using group-level observations, *z = 2*.*289*, *p = 0*.*022*).

It is noteworthy that the reported coefficients of variation measure relative variation in contributions by treatment using independent group-level observations. Thus, the reported coefficients of variation include contributions by rich and poor participants. We provide a more dis-aggregated analysis of variation in contributions in the Online Appendix D (Table A7) in S1 Appendix and report coefficients of variation for rich and poor (first- and second-movers) participants by treatment. These results show that the dispersion of contributions is higher in treatments with strong endowment inequality among rich and poor participants than in treatments with weak endowment inequality. There are no differences in the dispersion of contributions between different player types. Overall, these observations suggest that both types of players are more volatile in their contribution choices in treatments with strong inequality than in treatments with weak inequality. Taken together, we find that observed contribution patterns are more scattered under strong inequality than under weak inequality.

**Fig 6:**
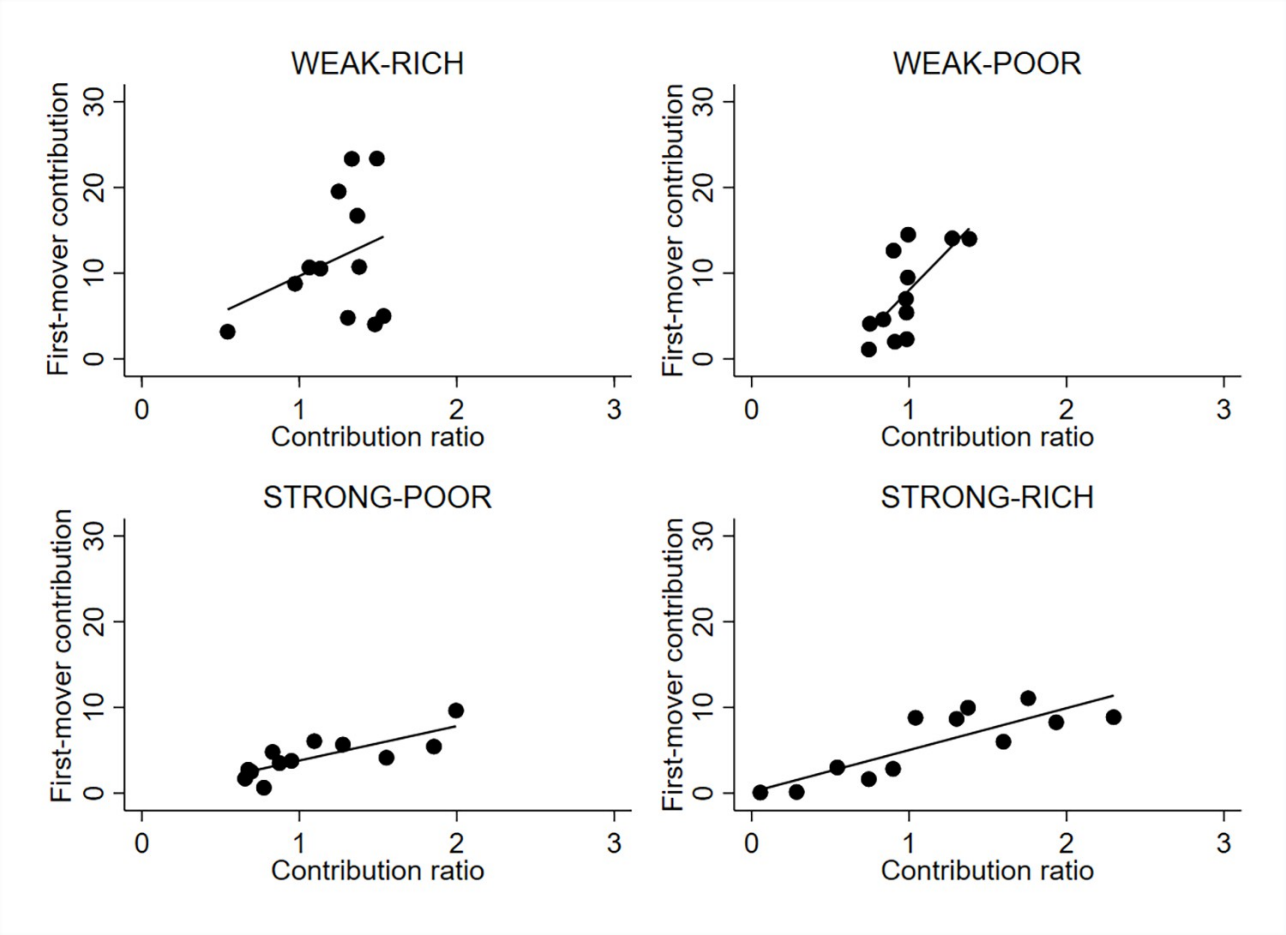
Scatter plots showing a relationship between the levels of first-mover contributions and observed second-mover contribution ratios by treatment. Scatter plots are drawn using group level observations. Solid lines show linear regression slopes.

To provide a more nuanced picture of the relationships between the first- and second-mover contributions, Fig 6 shows associations between the absolute levels of first-mover contributions and observed second-mover contribution ratios by treatment. First, we observe that the second-mover contribution ratios are clearly positively correlated with the levels of the first-mover contributions (Pearson’s Correlation coefficients: WEAK-RICH = 0.326, *p = 0*.*301*, WEAK-POOR = 0.693, *p = 0*.*013*, STRONG-RICH: 0.833 *p = 0*.*001*, STRONG-POOR: 0.793, *p = 0*.*002*). Second, Table 4 shows that the slopes of the regression lines are steeper in treatments with weak inequality than in treatments with strong inequality, suggesting that the levels of first-mover contributions are associated with greater increase in contribution ratios in weak inequality treatments than in strong inequality treatments.

**Table 4:** Predictors of contribution ratios (group level observations).

	Contribution ratio
First-mover-Average contribution	0.139*** (0.019)
WEAK-Treatment	0.468*** (0.156)
First-mover-Ave. contribution * WEAK-Treatment	-0.118*** (0.021)
R^2^	0.582
Observations	48

Notes: This table reports OLS regression coefficients using first-mover average contributions and group level contribution ratios (standard errors in parentheses). First-mover–average contribution denotes the average of first-mover contributions aggregated at the group level. WEAK-Treatment denotes an indicator variable that takes value 1 if the observations are from treatments WEAK-RICH or WEAK-POOR. The third predictor is an interaction term between these two variables. *** p < 0.001

**Result 3** *Contribution rules are more scattered under strong inequality than under weak inequality*.

### 4.3 Inequality and cooperation

**Fig 7:**
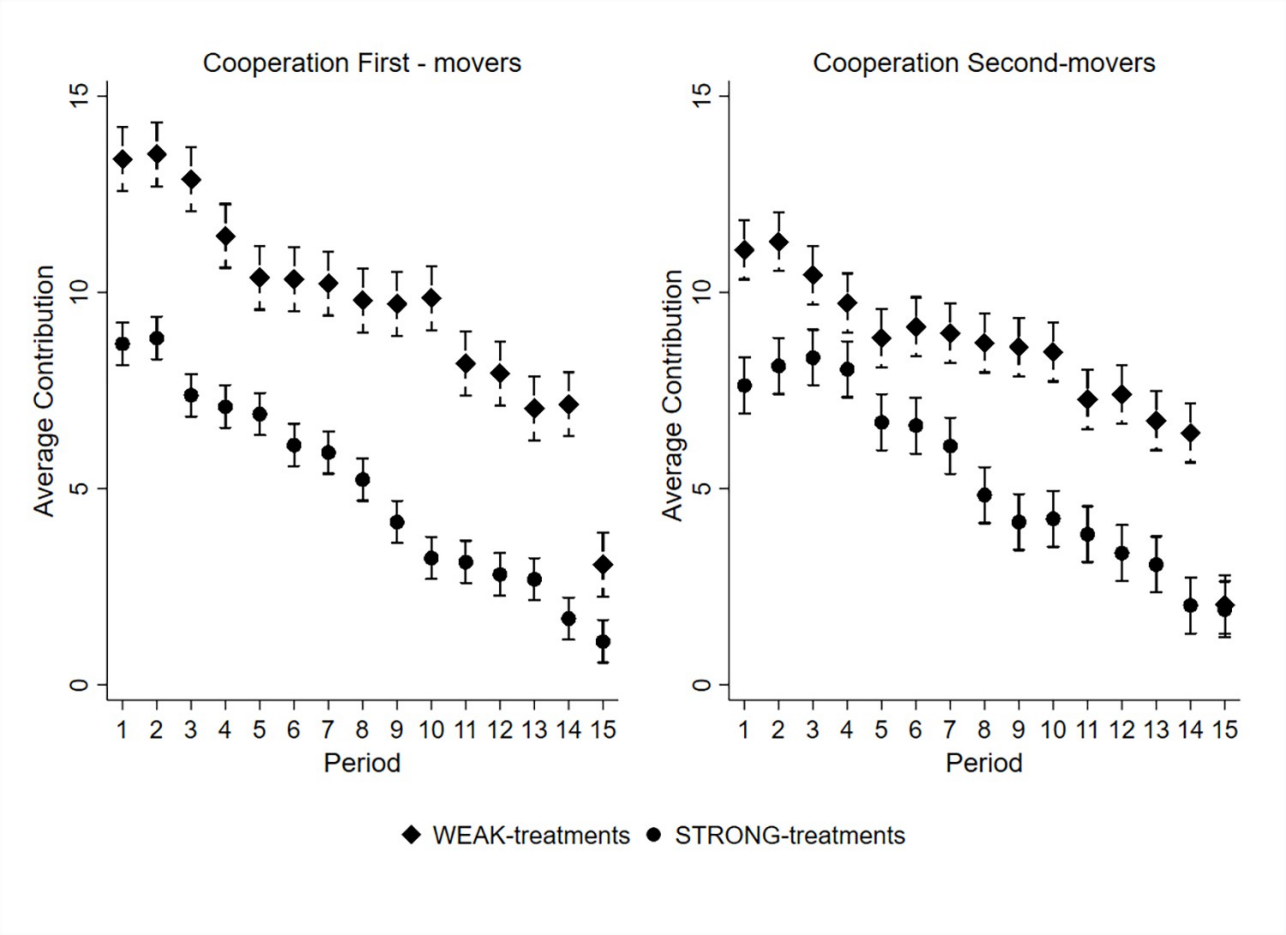
Average contributions (95% confidence intervals) by first- and second-movers by treatment.

**Fig 8:**
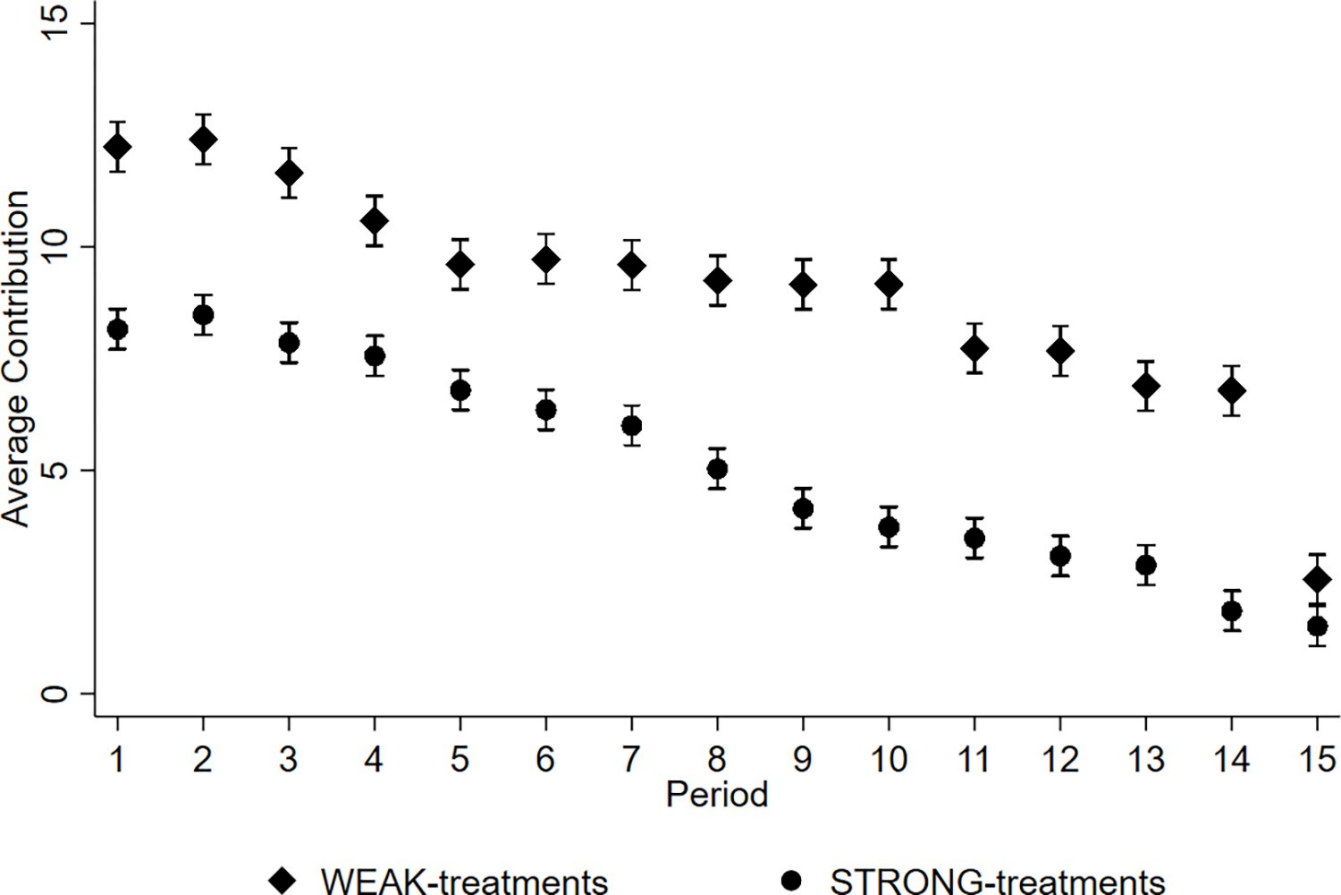
Average contributions (95% confidence intervals) over time by treatment.

Fig 7 illustrates the evolution of average contributions over time by first- and second-movers in weak and strong inequality treatments. Fig 8 shows the evolution of average contributions over time in weak and strong inequality treatments. We observe a strong decline in average contributions over time among first- and second-movers. This finding is consistent with voluminous literature on public good provision in finitely repeated games [45, 46].

#### 4.3.1 Average contributions

Table 5 summarizes average contributions to the group account across all periods by treatment. The average contribution in WEAK treatments is 9.0 ECUs and in STRONG treatments 5.1 ECUs. Thus, contributions in WEAK treatments are 76.5 percent higher than in STRONG treatments. The standardized effect size is 0.55 standard deviations. We find that average contributions are significantly higher in WEAK treatments than in STRONG treatments (Two-sample Wilcoxon rank-sum test performed on the aggregated average group contribution levels, *z = 2*.*330*, *p = 0*.*020*). The average contribution in WEAK treatments in the first period is 12.3 ECUs and in STRONG treatments 8.2 ECUs (Two-sample Wilcoxon rank-sum test using individual-level first period contributions: z = 4.409, p < 0.001, see Online Appendix D (Table A8) in S1 Appendix for first period contributions by treatment and endowment). The average contribution in WEAK treatments in periods from 1 to 5 is 11.3 ECUs and in STRONG treatments 7.8 ECUs (Two-sample Wilcoxon rank-sum test performed on the aggregated average group contributions from periods 1 to 5, *z = 2*.*052*, *p = 0*.*040*, see Online Appendix D (Table A9) in S1 Appendix for period 1 to 5 contributions by treatment and endowment). Thus, we observe that the difference between the WEAK and STRONG treatments is noticeable already in the first period. Taken together, we observe that individuals in strong inequality treatments are less cooperative than individuals in weak inequality treatments.

**Table 5:** Average contributions and pay-offs by treatment and player type.

Treatment	Contribution			Pay-off		
	Total	Rich	Poor	Total	Rich	Poor
WEAK-RICH	9.9 (5.5)	11.7 (7.3)	8.1 (4.1)	25.9 (3.3)	29.1 (2.0)	22.8 (5.1)
WEAK-POOR	8.1 (6.3)	8.6 (8.0)	7.6 (5.1)	24.9 (3.8)	29.4 (2.7)	20.4 (5.4)
STRONG-RICH	4.8 (2.7)	5.8 (4.0)	3.8 (2.0)	23.3 (3.0)	31.9 (1.2)	13.8 (2.9)
STRONG-POOR	5.5 (5.0)	6.7 (8.1)	4.2 (2.4)	22.9 (1.6)	32.0 (0.8)	14.6 (6.0)

Notes: Average contributions and average income by treatment and participant type. Standard deviations using group averages as observation units in parentheses.

We documented in Section 4.2 that observed behavioral patterns are more ambiguous in treatments with strong inequality than in treatments with weak inequality. Moreover, there is more support for equal relative contributions in weak inequality treatments than in strong inequality treatments. In the following, we analyze how these changes in contribution ratios are related to contribution levels among the rich and poor individuals. Table 5 (columns RICH and POOR) shows average contributions by initial endowment and treatment. We make following observations. First, we find that rich participants contribute less than poor participants in proportion to their initial endowment in treatments with strong inequality, but not in treatments with weak inequality (two-sided Wilcoxon rank-sum tests using group-level observations for WEAK-RICH: *z = 0*.*462*, *p = 0*.*644*, WEAK-POOR: *z = 1*.*270*, *p = 0*.*204*, STRONG-RICH: *z = 2*.*830*, *p = 0*.*005*, STRONG-POOR: *z = 2*.*425*, *p = 0*.*015*). Second, we observe a striking behavioral pattern between WEAK and STRONG treatments among rich participants. We find that the contributions by rich participants are significantly lower in absolute terms in STRONG treatments than in WEAK treatments. The average contribution by rich participants is 10.2 ECUs in WEAK treatments and 6.3 ECUs in STRONG treatments (two-sided Wilcoxon rank-sum test using group-level observations, *z = 1*.*980*, *p = 0*.*048*). Moreover, we find that the contributions by rich participants are lower in absolute terms in STRONG treatments than in WEAK treatments already in the first period. The average contribution by rich participants in the first period is 13.9 ECUs in WEAK treatments and 10.5 ECUs in STRONG treatments (two-sided Wilcoxon rank-sum test using first-period observations, *z = 2*.*044*, *p = 0*.*041*). This observation suggests that rich participants in STRONG treatments may anticipate the erosion of contribution rules and difficulty of cooperation under strong endowment inequality.

#### 4.3.2 Regression analyses

Table 6 reports mixed effects panel regressions with random effects at the individual and group levels to account for the dependencies between an individual’s contributions across periods and within group. The dependent variable is a participant’s contribution to the group account in a period.

**Table 6:** Mixed effect regressions of individual contributions with random effects at the individual and group levels.

	Contributions						
	Total			Rich		Poor	
	(1)	(2)	(3)	(4)	(5)	(6)	(7)
WEAK	3.873*** (1.453)	1.083** (.494)	1.739** (.734)	3.904** (1.995)	1.360 (1.345)	3.842*** (1.011)	1.980*** (.489)
Others’ contribution—l		0.227*** (.008)	0.177*** (.011)		0.212*** (.021)		0.153*** (.012)
Female			-0.564 (.517)		0.409 (0.661)		-0.661 (.356)
Age			-0.117** (.058)		-0.110* (.060)		-0.018 (.043)
Periods							
6–10			-0.932*** (.304)		-1.072*** (.046)		-0.728*** (.254)
11–15			-2.519*** (.374)		-2.879*** (0.505)		-2.031*** (.380)
Constant	5.127*** (.818)	1.244*** (.298)	6.441*** (1.542)	6.258*** (1.262)	6.673*** (1.604)	3.996*** (.432)	2.893*** (1.185)
Observations	2880	2688	2688	1440	1344	1440	1344

Notes: This table reports regression coefficients from mixed effects regressions with random effects at individual and group levels using individual-level data (robust standard errors adjusted for 48 groups in parentheses). Variable WEAK denotes an indicator variable that takes value 1 if the observations are from treatments WEAK-RICH or WEAK-POOR. Variable Others’ contribution—l denotes lagged (one-period) total contributions by the three other group members. Variable Period denotes indicator variables for periods 6–10 and 11–15 and shows their difference in contrast to an omitted indicator variable (Periods 1–5).

*** p < 0.001

** p < 0.005

Consistent with the non-parametric tests, Columns (1)–(3) in Table 6 show that the average contributions are significantly higher in WEAK treatments than in STRONG treatments. Columns (2) and (3) show, in line with a large literature on public goods games [46], that individuals’ condition their behavior on the (lagged) contributions of other group members. Columns (3), (5) and (7) document a non-linear declining time trend in contributions. Column (4) reiterates the finding that average contributions by rich participants are significantly lower in absolute terms in STRONG treatments than in WEAK treatments using a regression framework with random effects at the individual and group levels. Column (5), however, shows that adding the (one-period) lagged total contributions of other three group members to the regression model substantially reduces the magnitude of the WEAK treatment coefficient and renders it statistically insignificant. This observation suggests that the effect of the treatment manipulation on rich participants’ absolute contribution levels is overshadowed by the influence of their peers’ past contributions. Columns (6) and (7) document that average contributions by poor participants are significantly lower in absolute terms in STRONG treatments than in WEAK treatments.

**Result 4** *Voluntary contributions are lower in strong inequality treatments than in weak inequality treatments*.

## 5. Conclusion

This paper presents new evidence about the causal effects of endowment inequality on people’s perceptions of fairness and willingness to cooperate. Using experimental and survey data, we distinguish people’s injunctive perceptions of fairness from observed contribution patterns. We obtain four findings. First, we find that impartial observers hold shared normative views of fair contribution rules. The most appealing injunctive contribution principle is the equity principle, despite the differences in individual resources and levels of inequality. Second, individuals in the experiment, with their own money at stake, hold conflicting views over fair contribution rules in treatments with weak inequality. Observed contribution patterns in weak inequality treatments are consistent with a self-serving interpretation of fairness principles. Third, we observe that contribution patterns are more scattered under strong inequality than under weak inequality. Fourth, the erosion of commonly shared contribution patterns under strong inequality is associated with low levels of cooperation.

The plurality of plausible fairness principles is receiving growing attention as a key factor in economic decision-making. Consistent with the existing empirical literature, we find evidence that individuals hold conflicting perceptions of fairness. Moreover, we document a relationship between the erosion of commonly shared contribution patterns and low levels of cooperation under high endowment inequality. These observations are largely consistent with a view that normative disagreement induces individuals to put more weight on their selfish motives through self-serving bias and reduces contributions to common causes [44, 47].

Our paper is complimentary to studies that have focused on the effects of heterogeneous resource distributions on normatively appealing contribution rules [36, 44, 48, 49]. However, our study focuses explicitly on testing how individuals and groups behave under diverse levels of inequality. Our results complement the existing literature on normative conflict and suggest that greater resource inequality may lead to more disperse contribution rules. Moreover, our results suggest that there are systematical differences between the normative views of impartial external observers and observed behavioral patterns. Consequently, the comparison of injunctive perceptions of fairness and observed contribution patterns highlights the importance of data collection procedure (survey data vs. incentivized experiment) when motivating theoretical models of normative behavior with empirical observations.

We note that our paper is limited to comparing people’s perceptions of fairness and willingness to cooperate in groups with weak and strong inequality and does not aim to contribute to the debate how experiencing different degrees of inequality affects behavior. Complementary to our paper, Ramalingam and Stoddard [50] use a public goods experiment to study how individuals and groups react to changes in inequality and find that eliminating inequalities does not raise cooperation levels in groups that have experienced inequality in the past.

We acknowledge that our study has several limitations. Our outcome variables were measured using a laboratory experiment and online survey. The artificiality of the decision environment, experimenter demand effects and relatively modest monetary incentives may lead to distorted choices that do not generalize to naturally-occurring environments. Moreover, we note that the individuals participating in our experiment are mainly students from a Western, educated, industrialized, rich and democratic society [51]. Thus, our results may not generalize to other local and global samples with different social and demographic characteristics.

Our work investigates common patterns of behavior under varying levels of endowment inequality but is silent about the cognitive mechanisms that produce these patterns. While we can only speculate about the cognitive mechanisms that lead individuals to establish certain contribution rules in our experimental setup, a growing body of literature suggests that so-called dual system models, which distinguish between intuitive and deliberative decision-making, may help advance our understanding of cooperation in social dilemmas [52, 53]. It seems possible that the decision-processes in our experiment also contain two separate components: a more intuitive lump-sum deviation due to the unequal endowments and a more deliberative set of conditionally cooperative contribution strategies. Overall, it remains an open question how cognitive-processing manipulations that encourage intuitive decision-making, such as time pressure manipulations, would affect peoples’ fairness perceptions and behavior in public goods games with heterogenous endowments.

Our results raise new questions and directions for future research. A natural next step toward under- standing the causal effects of economic inequalities on the development of social capital, people’s fairness perceptions and willingness to cooperate is to design experiments that enable exogenous variation in resource inequality and outcome measurements in naturally-occurring environments. Measures of social capital and willingness to cooperate may be based on administrative and survey data, for example, on voluntary community participation, participation in charitable work and resource contributions to various cultural activities, hobby clubs, sports teams, residents’ associations, and educational institutions. Moreover, it is crucial to understand how the diverse sources of financial inequality and legitimacy of inequality interact with people’s perceptions of fairness and willingness to cooperate. Overall, emerging evidence about the predictability of preferred normative principles and self-interest use of equity arguments among individuals and social groups suggest new research agendas to examine how systematic differences in normative views of fair behaviors between individuals and social groups could be used to design novel institutional solutions to avoid the destructive nature of normative disagreement and foster cooperation in heterogeneous populations.

## Supporting information

S1 Appendix (PDF)
