# Supplementary material for: Fairness views and cooperation under varying levels of economic inequality
Source: PLoS One. 2023 Nov 9;18(11):e0288790. doi: 10.1371/journal.pone.0288790 (PMC10635489; doi:10.1371/journal.pone.0288790)
Supplement: S1 Appendix — (PDF) [file pone.0288790.s001.pdf]

Online appendix for

Fairness views and cooperation under varying levels of economic inequality

Wasilios Hariskos Jakob Neitzel Lauri Sääksvuori*

Contents

A Normative Principles **2**

A.1 Categorization rules 2

A.2 Categorization results 3

B Payoff Inequality **4**

C Supplementary Figures **5**

D Supplementary Tables **10**

E Mathematical Appendix **13**

F Experimental Instructions **16**

*Hariskos: University of Erfurt, Center for Empirical Research in Economics and Behavioral Sciences (CEREB), Nordhäußer Strasse 63, 99089 Erfurt, Germany, wasilios.hariskos@uni-erfurt.de. Neitzel: University of Hamburg, Department of Economics, Von-Melle-Park 5, 20146 Hamburg, Germany, jakob.neitzel@wiso.uni-hamburg.de. Sääksvuori: University of Turku and Finnish Institute for Health and Welfare, Centre for Health and Social Economics. University of Turku, INVEST Research Flagship Center, Assistentinkatu 7, 20014 University of Turku, Finland, lauri.saaksvuori@utu.fi.

A Normative Principles

A.1 Categorization rules

This section complements our analyses based on contribution ratios (Section 4.1) by categorizing participants' survey answers into different normative principles. Our primary interest pertains to normative principles that relate individual contributions to initial differences in endowments. The normative principle of *equity* suggests the equalization of absolute contributions. By contrast, the normative principle of *equity* links contributions to the initial endowments in a proportional manner and suggest the equalization of relative contributions. Yet, to provide a more comprehensive picture about participants' perceptions of fairness, we include the principles of *efficiency* and *equal payoffs* in our analyses. Including these alternative normative principles enables us to evaluate if we are likely to miss some key insights once concentrating on equality and equity principles.

We refer to the efficiency principle if a participant reports that the fairest possible contribution is the efficiency maximizing contribution. The principle of equal payoff refers to answers that imply the equalization of payoffs. Consequently, the principle of equal payoffs is defined as a contribution that completely closes the earnings gap between rich and poor participants due to initial differences in endowments.

Table A1 shows the applied categorization rules. Column *Uncond* refers to the unconditional questions in which participants were asked to simultaneously state unconditional contribution for poor and rich players. Columns *Rich* and *Poor* refer to the questions about conditional contribution among rich and poor players. Note that answers prescribing full contributions in the unconditional questions may be classified into three categories: *equity*, *efficiency* and *equal payoff*. In such cases, participants' answers are allocated to efficiency as the most restrictive contribution rule. We define the most restrictive contribution rule as a rule that comprises least combinations of contributions.

Table A1: Categorization rules using strict categorization

	WEAK			STRONG		
	Uncond	Rich	Poor	Uncond	Rich	Poor
Equity	$c_R = \frac{5}{3}c_P$	15	5	$c_R = 3c_P$	27	3
Equality	$c_R = c_P$	9	9	$c_R = c_P$	9	9
Efficiency	$c_R + c_P = 40$	25	15	$c_R + c_P = 40$	30	10
Equal Payoff	$c_R = c_P + 10$	19	0	$c_R = c_P + 20$	29	0

Notes: Reference contribution was set to 9 in all questions eliciting conditional contributions. For Equity, we use rounded values for the classification of answers. c_R: contribution by rich participants. c_P: contribution by poor participants.

To study the robustness of our classification algorithm and allocate a larger share of survey answers to the four fairness principles, we report results using a complementary categorization algorithm that allows for small decision errors of ± 1. Table A2 shows the exact categorization rules after allowing for decisions errors. In accordance with the previously described practice to provide the most conservative distribution of perceived fairness principles, we always classify answers to the most restrictive rule whenever two or more rules conflict. Under strong wealth inequality, we also account for the fact that contributions by rich participants required for *efficiency* and *equal payoffs* are not sufficiently distinct to allow for errors. The same issue arises for poor player contributions required for *efficiency* and *equality*. In these cases, the allocation is done as in the strict categorization.

Table A2: Categorization rules that allow small errors

	WEAK			STRONG		
	Uncond	Rich	Poor	Uncond	Rich	Poor
Equity	$c_R = \frac{5}{3}c_P \pm 1$	[14,16]	[4,6]	$c_R = 3c_P \pm 1$	[26,28)	[2,4]
Equality	$c_R = c_P \pm 1$	[8,10]	[8,10]	$c_R = c_P \pm 1$	[8,10]	[8,9]
Efficiency	$c_R + c_P \geq 38$	[24,25]	[14,15]	$c_R + c_P \geq 38$	(29,30]	(9,10]
Equal Payoff	$c_R - c_P \in [9, 11]$	[18,20]	[0,1]	$c_R - c_P \in [19, 21]$	[28,29]	[0,1]

Notes: Reference contribution was set to 9 in all questions eliciting conditional contributions For Equity, we use rounded values for the classification of answers. c_R: contribution by rich participants. c_P: contribution by poor participants.

A.2 Categorization results

Table A3 summarizes participants' opinions about fair contributions under weak and strong wealth inequality. Table A3(a) presents the fraction of answers corresponding to the normative contribution principles using the strict categorization rules defined above, while Table A3(b) displays the fraction of answers corresponding to the normative contribution principles according to the categorization that allows for small decision errors.

Using the strict categorization, we observe that the simple majority of classifiable opinions coincides with the equity norm in all questions. Simultaneously, we observe that we are able to allocate a fairly modest portion of answers to any clearly defined contribution principle. Using the less restrictive categorization rules that allow for small errors, the number of unclassified answers declines substantially. The vast majority of allocated answers are classified to the normative principle of equity. As a result of the looser categorization, the normative principle of equity is considered to be the fair contribution rule by the absolute majority of impartial observers in all cases but for rich players under strong inequality.

Table A3: Fraction of answers corresponding to normative fairness principles

(a) Strict categorization

	WEAK			STRONG		
	Uncond	Rich	Poor	Uncond	Rich	Poor
Equity	.24	.36	.45	.36	.32	.52
Equality	.03	.02	.01	.01	.02	.04
Efficiency	.12	.05	.04	.11	.06	.01
Equal Payoff	.15	.10	.05	.03	.09	.12
Other	.46	.47	.45	.49	.51	.31

(b) Categorization allowing for small errors

	WEAK			STRONG		
	Uncond	Rich	Poor	Uncond	Rich	Poor
Equity	.62	.56	.75	.60	.34	.75
Equality	.04	.07	.04	.01	.06	.04
Efficiency	.12	.05	.04	.11	.06	.01
Equal Payoff	.15	.17	.09	.03	.13	.16
Other	.07	.15	.08	.25	.41	.04

Notes: Numbers in each cell show the fraction of answers that correspond with the contribution principle. The category *Other* contains observation than cannot be classified to any normative principle using the definitions described in Tables A1 and A2.

B Payoff Inequality

In our decision setting, voluntary contributions to a public good may tighten or widen the inequality gap. This section investigates inequality between participants before and after voluntary contributions to the public good. Our primary measure of inequality is the Gini coefficient. The initial endowments for rich and poor participants in treatments with weak and strong wealth inequality are fixed. The Gini coefficient for differences in initial endowments before the public goods game is 0.125 in weak inequality treatments and 0.25 in strong inequality treatments.

Figure A1 illustrates Lorenz curves that show the distribution of income between the group members by treatment. The 45 degree line represents perfectly equal income levels. Table 2 shows the average pay-off among the rich and poor individuals by treatment. We observe that the ex-post pay-off inequality between rich and poor participants is smaller than the inequality in endowments due to higher absolute contributions by rich individuals. At the same time, the changes in inequality do not appear to vary between treatments. We test this qualitative observation as follows. First, we compute the Gini coefficient for pay-off inequality in every group after contributions to the public good. Second, we compute the difference between the ex-post (after the public goods contributions) and ex-ante (before the public

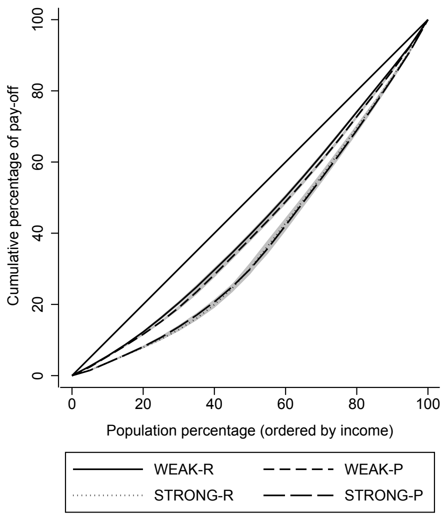

Figure A1: Lorenz curves by treatment

goods contributions) inequality in every group. Finally, we test whether the differences between the ex-post and ex-ante inequality differ between weak and strong inequality treatments. We find that the level of income inequality stays unchanged between the weak and strong treatments (two-sample Wilcoxon rank-sum test: $z = -0.351, p = 0.7259$). There is also no difference between the rich first-mover and poor first-mover treatments (two-sample Wilcoxon rank-sum test: $z = 1.402, p = 0.1609$).

C Supplementary Figures

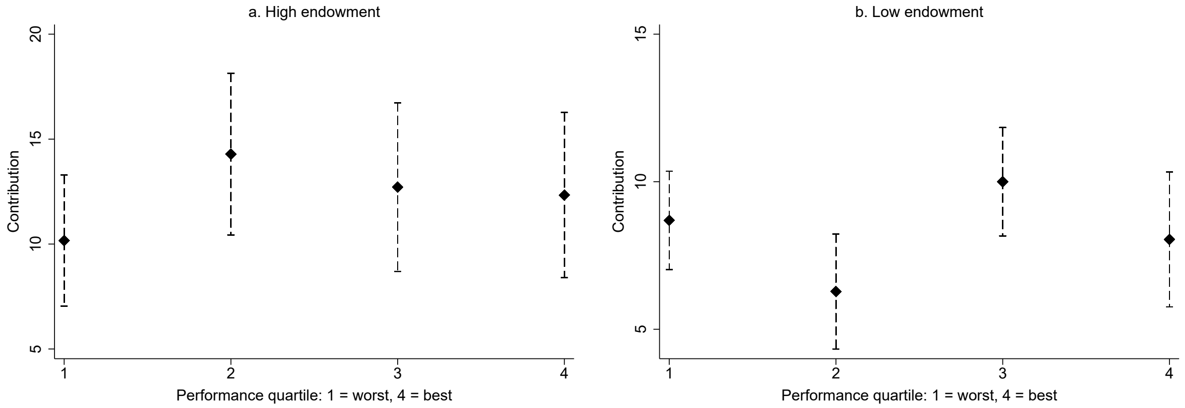

Figure A2: Public good contributions by performance in the real-effort task.

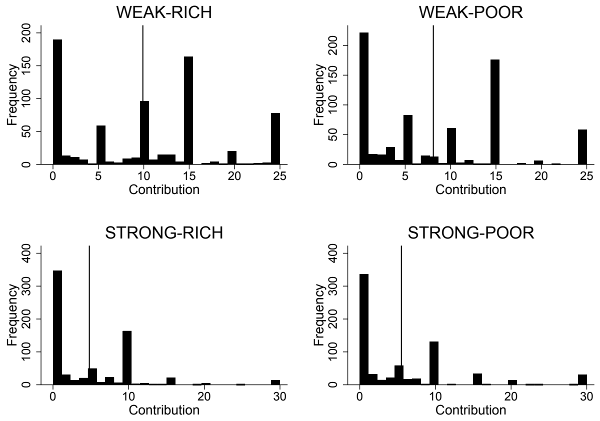

Figure A3: Histograms of all absolute contributions by treatment using individual level observations and all periods. Vertical lines display average contributions by treatment.

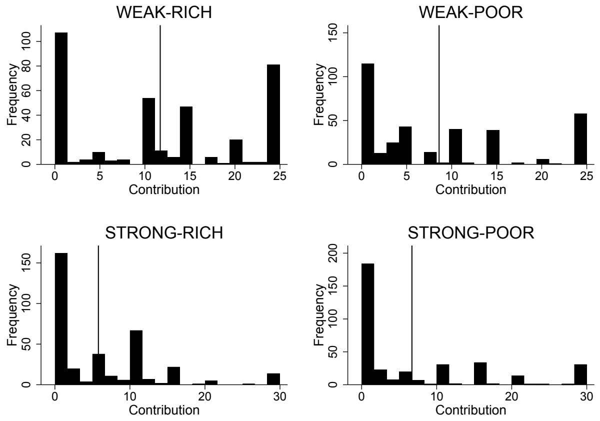

Figure A4: Histograms of rich participants absolute contributions by treatment using individual level observations and all periods. Vertical lines display average contributions by rich players by treatment.

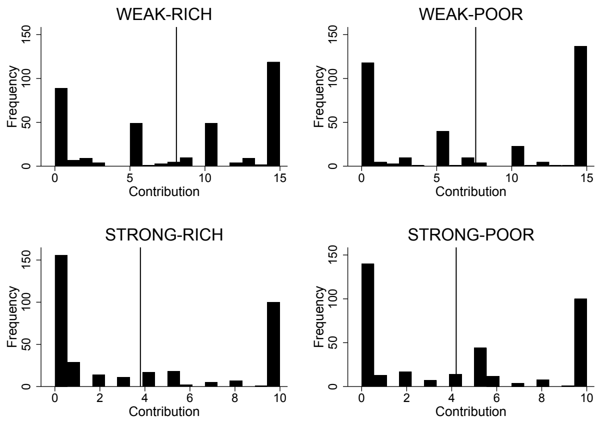

Figure A5: Histograms of poor participants absolute contributions by treatment using individual level observations and all periods.Vertical lines display average contributions by poor players by treatment

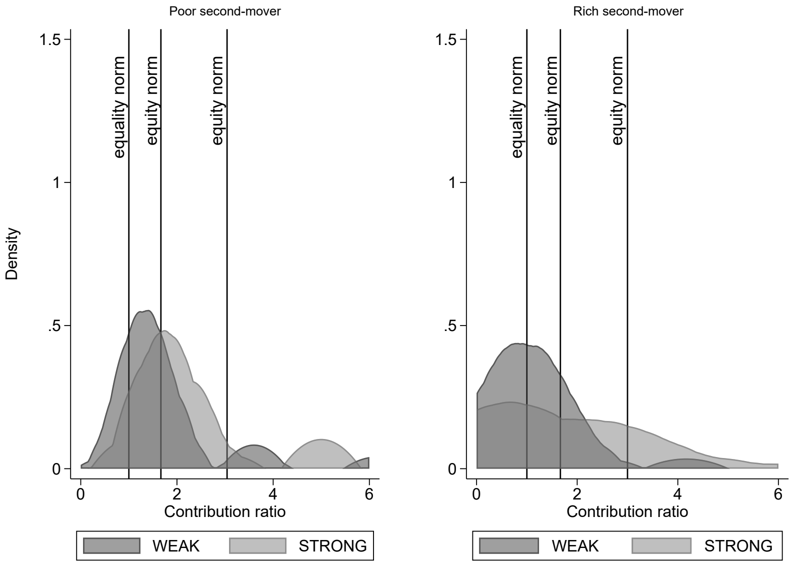

Notes: Kernel densities of average contribution ratios using the Epanechnikov kernel function. Bandwidths are calculated to minimize the mean integrated squared error for the underlying Gaussian density

Figure A6: The distribution of observed contribution ratios using only first period observations by poor and rich individuals under weak and strong endowment inequality.

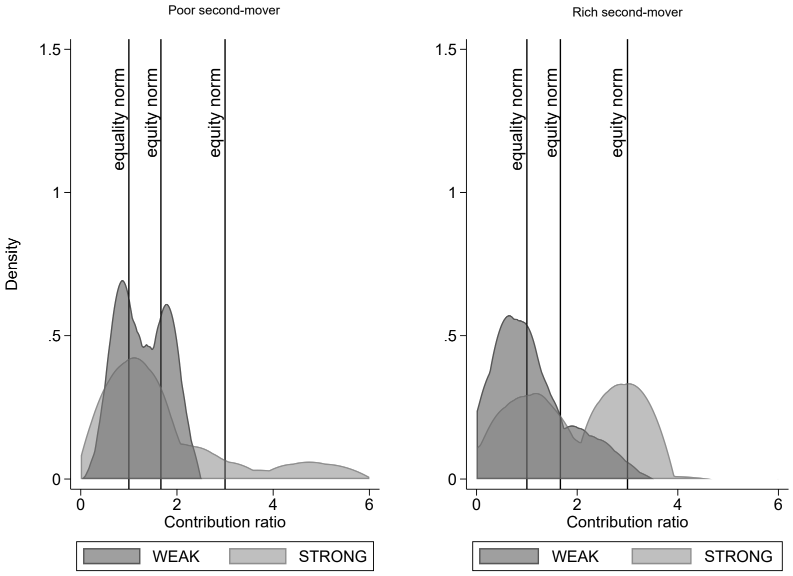

Notes: Kernel densities of average contribution ratios using the Epanechnikov kernel function. Bandwidths are calculated to minimize the mean integrated squared error for the underlying Gaussian density

Figure A7: The distribution of observed contribution ratios by poor and rich individuals under weak and strong endowment inequality using observations where the first-mover average contribution is 8 - 10 (9 ± 1).

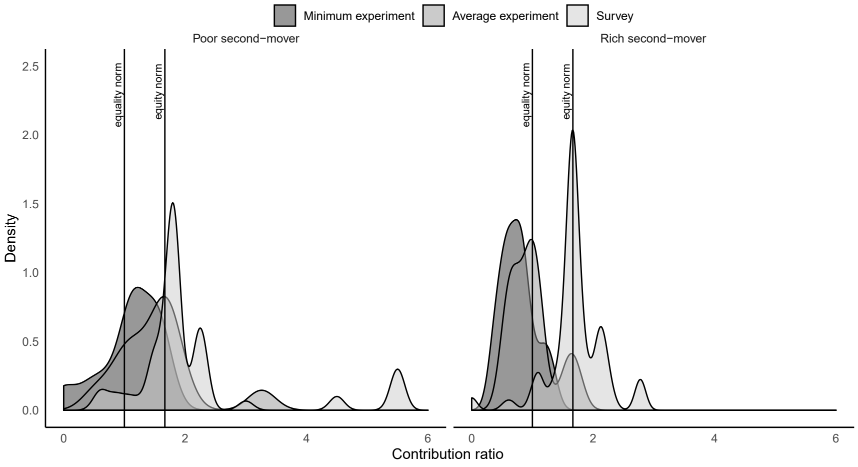

Notes: Kernel densities of average contribution ratios using the Epanechnikov kernel function. Bandwidths are calculated to minimize the mean integrated squared error for the underlying Gaussian density

Figure A8: The distribution of observed contribution ratios by poor and rich individuals under weak endowment inequality.

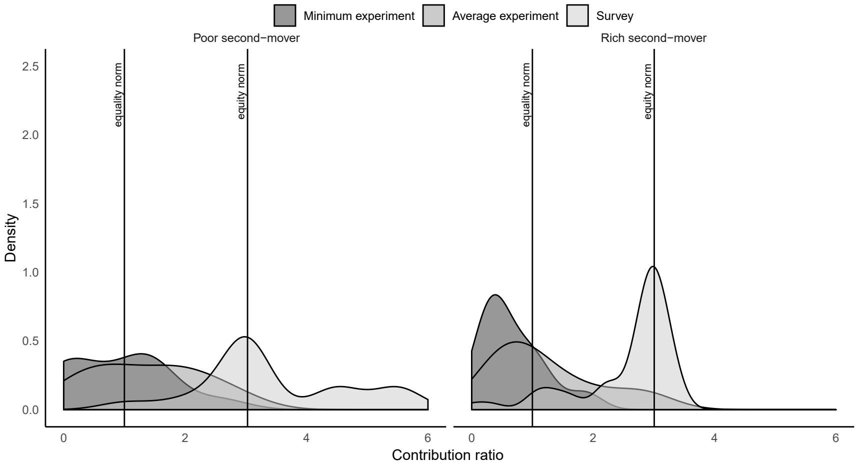

Notes: Kernel densities of average contribution ratios using the Epanechnikov kernel function. Bandwidths are calculated to minimize the mean integrated squared error for the underlying Gaussian density

Figure A9: The distribution of observed contribution ratios by poor and rich individuals under strong endowment inequality.

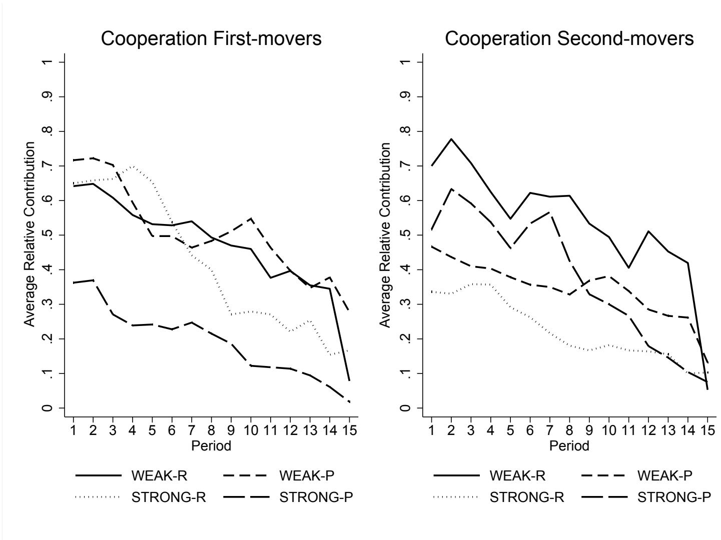

Figure A10: Average relative contributions by first- and second-movers by treatment.

D Supplementary Tables

Table A4: Participants' answers to unconditional survey questions under weak inequality.

	0	0.3	3	4	5	6	7	7.5	8	10	12	15
0.4		1										
5	1		2	1	2							
7			1	2								
8				1	1							
9					1							
10	1				22	3	1					
12					2		10		2			
12.5								3				
13							1		1			
15							3			8		1
18									1		1	
19												1
20										6	1	
25												12

Notes: Table shows the cross-tabulation of answers to unconditional survey questions under weak inequality. Columns represent suggested contributions for poor participants. Rows represent suggested contributions for rich participants. Each cell shows the number of suggested combinations of contributions. N=100

Table A5: Participants' answers to unconditional survey questions under strong inequality.

	0	1	2	3	4	5	6	7	10
5			1						
7				1					
8				1					
9				1					
10	2		2	12		1			1
11		1							
12			1		3				
13				1					
14					1				
15	1		1		1	31			1
16									
18						2	1		
20	2		1	1	3	9	1	1	1
25	1					1			1
30									11

Notes: Table shows the cross-tabulation of answers to unconditional survey questions under strong inequality. Columns represent suggested contributions for poor participants. Rows represent suggested contributions for rich participants. Each cell shows the number of suggested combinations of contributions. N=100

Table A6: Predictors of second-mover contributions

	Contribution								
	All Treatments			Rich-first Treatments			Poor-first Treatments		
	(1)	(2)	(3)	(4)	(5)	(6)	(7)	(8)	(9)
FM-Average	0.686		0.345	0.515		0.507	1.312		0.629
	(.022)		(.064)	(.017)		(.050)	(.044)		(.119)
	[.629]		[.316]	[.751]		[.740]	[.742]		[.356]
FM-Minimum		0.686	0.363		0.489	0.008		1.251	0.695
		(.022)	(.064)		(.018)	(.050)		(.042)	(.113)
		[.631]	[.334]		[.708]	[.012]		[.746]	[.415]
R^2	0.396	0.398	0.410	0.565	0.501	0.565	0.551	0.557	0.574
Observations	1440	1440	1440	720	720	720	720	720	720

Notes: This table reports regression coefficients for second-mover contributions (standard errors in parentheses). Squared brackets show standardized regression coefficients. *FM-Average* denotes first-mover average contribution. *FM-Minimum* denotes first-mover minimum contribution.

Table A7: Coefficients of variation by player type and treatment.

Treatment	Rich	Poor
WEAK-RICH	0.76	0.77
WEAK-POOR	0.89	0.98
STRONG-RICH	1.24	1.27
STRONG-POOR	1.46	1.11

Notes: Table shows coefficients of variation by player type and treatment. Label Rich refers to participants with high endowment. Label Poor refers to participants with low endowment.

Table A8: First period average contributions and pay-off by treatment and player type

	Contribution			Pay-off		
Treatment	Total	Rich	Poor	Total	Rich	Poor
WEAK-RICH	13.3	16.0	10.5	28.0	30.2	25.7
	(7.4)	(8.4)	(5.1)	(6.5)	(7.8)	(4.1)
WEAK-POOR	11.2	11.7	10.8	26.7	31.3	22.2
	(6.6)	(8.7)	(4.3)	(7.6)	(7.2)	(4.8)
STRONG-RICH	8.0	10.9	5.2	24.8	32.0	17.7
	(7.9)	(9.5)	(4.4)	(9.3)	(6.7)	(5.2)
STRONG-POOR	8.3	10.1	6.5	28.9	33.2	16.8
	(7.4)	(9.8)	(3.4)	(10.6)	(7.1)	(6.2)

Notes: First period average contributions and average income by treatment and participant type. Standard deviations using individual observations as observation units in parentheses.

Table A9: Average contributions from periods 1 - 5 by treatment and player type

	Contribution		
Treatment	Total	Rich	Poor
WEAK-RICH	12.5	14.9	10.1
	(5.4)	(6.6)	(4.5)
WEAK-POOR	10.1	10.5	9.7
	(5.5)	(7.3)	(4.0)
STRONG-RICH	8.3	10.0	6.7
	(4.8)	(7.8)	(2.2)
STRONG-POOR	7.2	8.9	5.5
	(4.7)	(6.8)	(3.1)

Notes: Average contributions from period 1 - 5 by treatment and participant type. Standard deviations using individual observations as observation units in parentheses.

E Mathematical Appendix

Proof of Conjecture 1

The optimal contribution level for maximizing utility function u_i results to

$$c_i(k, c_{-i}) = m(k, c_{-i}) - \frac{1-\alpha}{\beta}.$$

Since $k \in \{0, 1\}$, we need to compare the utilities that player i gets from applying the two alternative norms. Utility amounts to

$$u_i(\frac{c_{-i}}{n-1}, c_{-i}, 0) = w_i + c_{-i}(\alpha + (\alpha - 1)\frac{1}{n-1}) + \frac{(1-\alpha)^2}{2\beta}$$

when $k = 0$ and to

$$u_i(\frac{w_i}{w_{-i}}c_{-i}, c_{-i}, 1) = w_i + c_{-i}(\alpha + (\alpha - 1)\frac{w_i}{w_{-i}}) + \frac{(1-\alpha)^2}{2\beta},$$

when $k = 1$.

Consequently, we find that

$$u_i(\frac{c_{-i}}{n-1}, c_{-i}, 0) > u_i(\frac{w_i}{w_{-i}}c_{-i}, c_{-i}, 1) \Leftrightarrow w_i > \frac{w_{-i}}{n-1}$$

and vice versa

$$u_i(\frac{c_{-i}}{n-1}, c_{-i}, 0) < u_i(\frac{w_i}{w_{-i}}c_{-i}, c_{-i}, 1) \Leftrightarrow w_i < \frac{w_{-i}}{n-1}.$$

Hence, if the endowment of player i is larger than the average endowment of other players, she applies the equality norm. If the endowment of player i is lower than the average endowment of other players, the equity norm is applied.

Proof of Conjecture 2

Let h be the number of rich players and l the number of poor players. First, we consider the rich first-mover mechanism. In the case of weak endowment inequality, the sum of contributions results to

$$hxw_h + lxw_l = x\sum_i w_i.$$

Since the sum of endowments is kept constant, public good provision is equal for all possible distributions of weak endowment inequality. When crossing the threshold to strong endowment inequality ($w_l < xw_l$), both rich and poor players adjust their contributions downward. Rich contribute now w_l and poor contribute $\frac{w_l}{w_h}w_l < xw_l$, resulting into total contributions of

$$(h + l\frac{w_l}{w_h})w_l.$$

It is obvious that any further increases in endowment inequality decreases the total amount of public goods provision.

Second, let us consider the poor first-mover mechanism. Regardless of the degree of endowment inequality the total amount of public good provision results to

$$lxw_l + hxw_l = nxw_l.$$

Thus, with increasing endowment inequality and constant sum of endowments, contributions to the public good are decreasing.

Finally, let us consider the mixed sequence mechanism. Let $h^{'} \geq 1$ and $l^{'} \geq 1$ be the number of rich and poor first-movers. In the case of weak endowment inequality, the sum of contributions results to

$$h^{'}xw_h(1 + \frac{h - h^{'}}{h' + l'}) + xw_l(l + \frac{l^{'}(h - h^{'})}{h' + l'}).$$

Suppose that we increase the endowment of rich players by ϵ. The endowment of poor players has to decrease by $\frac{h}{h}\epsilon$. If there is still weak endowment inequality, new total contributions result to

$$h^{'}xw_h(1 + \frac{h - h^{'}}{h' + l'}) + xw_l(l + \frac{l^{'}(h - h^{'})}{h' + l'}) + h^{'}\epsilon x(1 + \frac{h - h^{'}}{h' + l'}) - \frac{h}{l}\epsilon x(l + \frac{l^{'}(h - h^{'})}{h' + l'})$$

Hence, total contributions increase if and only if

$$h^{'}\epsilon x(1+\frac{h-h^{'}}{h'+l'})-\frac{h}{l}\epsilon x(l+\frac{l^{'}(h-h^{'})}{h'+l'})>0$$

is true. We can derive that this condition requires $l^{'}(1+\frac{h}{l})<0$, which constitutes a contradiction to the assumption that $l^{'},h,l>0$. Therefore, total contributions are decreasing under increasing weak endowment inequality.

Now, assume that the threshold to strong endowment inequality is transcended. From that point onward, rich first-movers (contribution: w_l), poor first-movers (xw_l) and rich second-movers ($\frac{(h^{'}+l^{'}x)w_l}{h'+l'}$) all contribute less with increasing endowment inequality, since w_l is decreasing. Thus, it remains to be shown that also contributions of poor-second movers decrease when crossing the threshold from weak to strong endowment inequality. In the case of strong endowment inequality, poor second-movers contribute

$$\frac{(h^{'}+l^{'}x)w_l}{h'w_h+l'w_l^2}.$$

This function is increasing in w_l if the sum of endowments is kept constant and, vice versa, decreasing under constant overall sum of endowments if w_l is decreasing. To do so, we substitute w_h with $w_h=\frac{W}{h}-\frac{l}{h}w_l$, where W is the sum of endowments in the society. After simplifications, the derivative of the contribution function results to

$$\frac{w_l(h^{'}+l^{'}x)\frac{h^{'}}{h}(2W-lw_l)+l^{'}w_l)}{(\frac{h'}{h}(W-lw_l)+l'w_l)^2}$$

It is easy to see that the derivative is positive for all $w_l>0$, since $W>lw_l$. Hence, if w_l decreases, so does the contribution of poor second-movers. Thus, contributions are decreasing in increasing endowment inequality in the mixed order mechanism.

F Experimental Instructions

Instructions

Welcome and thank you for participating in this experiment. By carefully reading the instructions and depending on your decisions in the experiment you can earn a considerable amount of money. Your earnings will be privately paid to you in cash at the end of the experiment. The payment will be done in private, such that no other participant will know the amount of your earnings.

Please read these instructions carefully. They are solely for your private information. From now on, we ask you to remain seated and stop all communication with the other participants. Please switch off your mobile phone and remove all non-necessary items from your seat. If you have any questions at any time during the course of this experiment, please raise your hand and a member of the experimenter team will privately assist you. It is important that you follow these rules as any violation will lead to exclusion from the experiment and all payments.

During the experiment all decisions and transfers are made in Experimental Monetary Units (EMUs). At the end of the experiment, your income will be calculated in EMUs and converted to Euro at the following rate:

1 EMUs = 0.40 Euro

This experiment consists of two stages. You will complete the first stage before learning more about the decisions during the second stage. At the beginning of the first stage you will be randomly matched with three other participants in this room. That is, you will be part of a group of four people.

STAGE 1

All group members are given the same task. You will be presented with a number of words on your computer screen and your task will be to encode these words by substituting the letters with numbers using a conversion table presented on your computer screen. The same table is made available also on the bottom of this page. For all participants the sequential order of the presented words is the same.

Example: You are given the word SCHNITZEL. The letters in the Table show that S=1, C=7, H=2, N=18, I=8, T=12, Z=13, E=11, L=19.

Your performance during the task will determine your endowment size during the second stage. Your endowment will depend on the number of points you and the other participants in your group have after the encoding period of 10 minutes. Two persons with the highest numbers of encoded words will receive an endowment of 25 EMUs. Two persons with the lowest numbers of encoded words will receive an endowment of 15 EMUs. In other words, if you have encoded more words than at least two other

members in your group, you will receive an endowment of 25 EMUs. If you have encoded less words than at least two other members in your group, you will receive an endowment of 15 EMUs. You will not be told the exact number of words that the other group members have enconded and your exact rank within your group. Other group members will also nor receive information about the number of words you encoded. If two or more individuals have encoded the same number of words, the computer will randomly determine the ranking of the tied persons. Each person will have the same probability of being ranked above the other group members with the same amount of points.

A	B	C	D	E	F	G	H	I	J	K	L	M
8	12	14	10	9	6	24	22	7	5	11	3	18
N	O	P	Q	R	S	T	U	V	W	X	Y	Z
1	21	16	23	2	13	19	25	4	26	17	20	15

STAGE 2 [for treatment WEAK-STRONG]

The second stage of the experiment consists of 15 consecutive decision periods. In all 15 periods, you will make your decisions in the same group of four people that you were matched with at the beginning of the experiment. In other words, you are interacting with the same three people throughout the experiment.

As a result of Stage 1, there are two members in your group who are endowed with 25 EMUs and two members who are endowed with 15 EMUs. Your task is to decide how many EMUs you want to keep in your private account and how many EMUs you want to contribute to a group account. Each unit not allocated to the group account will be automatically remain in your private account.

Group members will make sequential decisions about the allocation of endowments to the private and group account. More precisely, there will be two early deciders and two late deciders. The late deciders will be informed about the decision of the early deciders before making their decisions. However, all participants will be informed about the decisions of all other group members at the end of the period. For that reason, every group member will be assigned an identity A, B, C or D. The two early deciders both have an endowment of 25 EMUs and identities A or B and the two late deciders have an endowment of 15 EMUs and identity C or D. In other words, if you have earned an endowment of 25 EMUs in Stage 1, you are an early decider. If you have earned an endowment of 15 EMUs, you are a late decider.

Your income from the private account

Each unit allocated to your private account earns you one EMU. No other member in your group benefits from transfers to your private account.

Income from private account = Your endowment - Your contribution to group account

Your income from the group account

For each EMU you contribute to the group account, you will earn 0.4 EMUs. All other members in your group will also earn 0.4 EMUs. Thus, the contribution of 1 EMU to the group account yields a total of 1.6 EMUs for all group members together. Your earnings from the group account are based on total number of EMUs contributed by all members in your group. Each group member will earn equally from the contributed amount. Thus, you earn both from your own contribution as well as from the contributions of all other group members. The income from the group account for all group members be determined by

Income from group account = Sum of contributions to group account $\cdot 0.4$

Your total income

Your total earnings result per period is the sum of your income from your private account and the income from the group account.

Total income = Earnings from your private account + Earnings from the group account

BONUS STAGE

In addition to the actual allocation decisions, we ask you to estimate the average contribution by the other three members in your group. You will make these guesses before the actual decisions are made.

You will be asked to estimate the contributions of group members with endowments 15 EMUs and 25 EMUs separately. In other words, if you have an endowment of 25 EMUs (15 EMUs), you estimate the contribution of the other group member with an endowment of 25 EMUs (15 EMUs) and the average contribution of the two group members with an endowment of 15 EMUs (25 EMUs). Please give your guesses in integers. If necessary, the average contribution will be rounded to the nearest integer. You will be rewarded for the accuracy of your estimates as follows:
(i) If your estimate exactly corresponds with the actual (average) allocation, you will earn 2 EMUs.
(ii) If your estimate deviates one EMU from the actual (average) allocation, you will earn 1 EMU.
(iii) If your estimate deviates two or more EMUs from the actual (average allocation), you will not earn any EMUs.

Hence, per period you can additionally earn uop to 4 EMUs through correct guesses.

Your total earnings at the end of the experiment

At the end of the experiment, one out of the 15 periods will be randomly chosen as relevant for payment for both total income and bonus. That is, the total income of one period will be paid out at the end. Therefore each period may be relevant for your earnings. The same is true for the bonus payments. The payment relevant period may be the same for income and bonus, but it does not have to be the same. Your total earnings therefore comprise of

Your total income in the randomly drawn period (converted into Euro)
+ Your earnings from the bonus stage in the randomly drawn period (converted into Euro)
= Your total earnings from the experiment

Questionnaire (online)

Welcome! Thank you for participating in this questionnaire study. Answering all questions will take about 10 minutes. If you complete the questionnaire, you will have a chance to win 100 Euros.There are 100 participants in this study. We will randomly draw two participants our of the 100 participants. The lucky winners will receive a cash prize of 100 Euros. Please enter your e-mail address at the end of the questionnaire. We will contact you by e-mail if you have been drawn as a winner.

This questionnaire consists of two parts. In the first part, we ask you to make familiarize yourself with a decision making problem. You will receive experimental instructions that describe an economics experiment. Please read the instructions carefully. However, you will not take part in this experiment, but act as a neutral external observer. The questions that you are asked to respond to will be explained more closely in the second part of the questionnaire.

PART 1

Please read carefully the following instructions that describe an economics experiment and answer the subsequent quiz questions. These questions are designed to help you understand the decision problem and to make sure that you have understood the instructions correctly. Once you have found the correct answer for a question, please enter it in the corresponding box. As soon as all questions have been answered correctly, you may proceed to the second part of this questionnaire.

[Experimental instructions as in the experiment]

PART 2

Scenario 25-15

In this scenario the two better encoders from stage 1 receive in stage 2 an endowment of 25 EMUs and the two worse encoders an endowment of 15 EMUs. We ask you to express your personal opinion about fair behavior in the previously described economics experiment. We ask you to express your opinion from the viewpoint of a neutral external observer. With fair behavior, we mean behavior that may be characterized as "socially appropriate" or "ethically correct".

[Screen 1] From the viewpoint of a neutral external observer, what is in your opinion a fair contribution to the group account by a group member who has an endowment of 25 ECUs? From the viewpoint of a neutral external observer, what is in your opinion a fair contribution to the group account by a group member who has an endowment of 15 ECUs?

[Screen 2a] A group member with an endowment of 15 EMUs has contributed 9 EMUs to the group account. From the viewpoint of a neutral external observer, what is in your opinion a fair contribution to the group account by a group member who has an endowment of 25 ECUs?

[Screen 2b] A group member with an endowment of 25 EMUs has contributed 9 EMUs to the group account. From the viewpoint of a neutral external observer, what is in your opinion a fair contribution to the group account by a group member who has an endowment of 15 ECUs?

Scenario 30-10

In this scenario the two better encoders from stage 1 receive in stage 2 an endowment of 30 EMUs and the two worse encoders an endowment of 10 EMUs. We ask you to express your personal opinion regarding fair behavior from the viewpoint of a neutral external observer. With fair behavior, we mean behavior that may be characterized as "socially appropriate" or "ethically correct".

[Screen 1] From the viewpoint of a neutral external observer, what is in your opinion a fair contribution to the group account by a group member who has an endowment of 30 ECUs? From the viewpoint of a neutral external observer, what is in your opinion a fair contribution to the group account by a group member who has an endowment of 10 ECUs?

[Screen 2a] A group member with an endowment of 10 EMUs has contributed 9 EMUs to the group account. From the viewpoint of a neutral external observer, what is in your opinion a fair contribution to the group account by a group member who has an endowment of 30 ECUs?

[Screen 2b] A group member with an endowment of 30 EMUs has contributed 9 EMUs to the group account. From the viewpoint of a neutral external observer, what is in your opinion a fair contribution to the group account by a group member who has an endowment of 10 ECUs?
